# IRF-3, IRF-5, and IRF-7 Coordinately Regulate the Type I IFN Response in Myeloid Dendritic Cells Downstream of MAVS Signaling

**DOI:** 10.1371/journal.ppat.1003118

**Published:** 2013-01-03

**Authors:** Helen M. Lazear, Alissa Lancaster, Courtney Wilkins, Mehul S. Suthar, Albert Huang, Sarah C. Vick, Lisa Clepper, Larissa Thackray, Margaret M. Brassil, Herbert W. Virgin, Janko Nikolich-Zugich, Ashlee V. Moses, Michael Gale, Klaus Früh, Michael S. Diamond

**Affiliations:** 1 Department of Medicine, Washington University School of Medicine, St. Louis, Missouri, United States of America; 2 Vaccine and Gene Therapy Institute, Oregon Health and Sciences University, Beaverton, Oregon, United States of America; 3 University of Washington School of Medicine, Seattle, Washington, United States of America; 4 Department of Pathology and Immunology, Washington University School of Medicine, St. Louis, Missouri, United States of America; 5 Department of Immunobiology and the Arizona Center on Aging, University of Arizona College of Medicine, Tucson, Arizona, United States of America; 6 Department of Molecular Microbiology, Washington University School of Medicine, St. Louis, Missouri, United States of America; Mount Sinai School of Medicine, United States of America

## Abstract

Although the transcription factors IRF-3 and IRF-7 are considered master regulators of type I interferon (IFN) induction and IFN stimulated gene (ISG) expression, *Irf3^−/−^×Irf7^−/−^* double knockout (DKO) myeloid dendritic cells (mDC) produce relatively normal levels of IFN-β after viral infection. We generated *Irf3^−/−^×Irf5^−/−^×Irf7^−/−^* triple knockout (TKO) mice to test whether IRF-5 was the source of the residual induction of IFN-β and ISGs in mDCs. In pathogenesis studies with two unrelated positive-sense RNA viruses (West Nile virus (WNV) and murine norovirus), TKO mice succumbed at rates greater than DKO mice and equal to or approaching those of mice lacking the type I IFN receptor (*Ifnar^−/−^*). In *ex vivo* studies, after WNV infection or exposure to Toll-like receptor agonists, TKO mDCs failed to produce IFN-β or express ISGs. In contrast, this response was sustained in TKO macrophages following WNV infection. To define IRF-regulated gene signatures, we performed microarray analysis on WNV-infected mDC from wild type (WT), DKO, TKO, or *Ifnar^−/−^* mice, as well as from mice lacking the RIG-I like receptor adaptor protein MAVS. Whereas the gene induction pattern in DKO mDC was similar to WT cells, remarkably, almost no ISG induction was detected in TKO or *Mavs^−/−^* mDC. The relative equivalence of TKO and *Mavs^−/−^* responses suggested that MAVS dominantly regulates ISG induction in mDC. Moreover, we showed that MAVS-dependent induction of ISGs can occur through an IRF-5-dependent yet IRF-3 and IRF-7-independent pathway. Our results establish IRF-3, -5, and -7 as the key transcription factors responsible for mediating the type I IFN and ISG response in mDC during WNV infection and suggest a novel signaling link between MAVS and IRF-5.

## Author Summary

Host pathogen sensors, including those of the Toll-like receptor and RIG-I like receptor (RLR) families, detect viral infection in cells. Signaling through these receptors triggers expression of type I interferon (IFN) and IFN-stimulated genes (ISGs), in part through the IRF family of transcription factors. Previous studies with West Nile virus (WNV) showed that IRF-3 and IRF-7 control IFN expression in fibroblasts and neurons, whereas macrophages and myeloid dendritic cells (mDC) retained the ability to induce IFN-β without IRF-3 and IRF-7. In the current study, we generated *Irf3^−/−^*×*Irf5^−/−^*×*Irf7^−/−^* (TKO) mice to characterize the contributions of specific IRF transcription factors to IFN and ISG induction in response to WNV infection in cells and in mice. We found that induction of IFN and ISGs was largely abolished in TKO mDC, but sustained in TKO macrophages. Because IFN and ISG induction also was absent in mDC lacking MAVS, a key mediator of RLR signaling, our results suggest a novel signaling link between IRF-5 and MAVS. This study establishes the molecular pathways responsible for IFN induction in mDC and suggests a cross-talk between IRF-5 and RLR signaling pathways.

## Introduction

The type I interferon (IFN) signaling network is an essential component of the innate immune response against viral infections, and also functions to shape adaptive immunity [1]–[4]. Infected cells initiate an antiviral response upon recognition of non-self pathogen-associated molecular patterns (PAMPs), which are detected by host pattern recognition receptors (PRRs) [2], . PRRs that recognize RNA viruses include members of the Toll-like receptor (TLR3 and TLR7) and the RIG-I-like receptor (RLR; RIG-I and MDA5) families. TLRs and RLRs recognize distinct PAMPs in different locations (extracellular/endosomes and cytoplasm, respectively) and activate signaling cascades to initiate antiviral and inflammatory responses. TLR3 binds to double-stranded RNA and recruits the adaptor molecule TRIF to activate the kinases TRAF and IKK-ε, which in turn activates the latent transcription factors IRF-3, IRF-7, and NF-κB. Single-stranded RNA is recognized by TLR7, which uses the adaptor molecule MyD88 to activate TRAF and IKK-ε, and subsequently NF-κB- and IRF-7-dependent transcription. RLRs interact with the mitochondria-associated adapter molecule MAVS (also called IPS-1, VISA, or CARDIF), which signals through the kinases TBK1 and IKK-ε to activate IRF-3, IRF-7, and NF-κB and initiate type I IFN production.

A canonical model for type I IFN production after RNA virus infection is a two-step positive feedback loop that is regulated by IRF-3 and IRF-7 [9], [10]. In the first phase, viral sensing by TLRs or RLRs induces nuclear localization of IRF-3, which in concert with NF-κB and ATF-2/c-Jun stimulates transcription, synthesis, and secretion of IFN-β and IFN-α4 by infected cells. In the second phase, extracellular IFN-β and IFN-α4 bind to the type I IFN receptor (IFNAR), which triggers activation of the JAK-STAT signaling pathway and induction of IFN-stimulated genes (ISGs) [11]. ISGs act by a variety of mechanisms to render cells resistant to viral replication [12], [13]. Although type I IFN signaling is required to activate the full antiviral response, a subset of ISGs is induced directly by IRF-3 [14], [15]. While IRF-3 is constitutively expressed in many tissues, IRF-7 is an ISG required for the expression of most IFN-α subtypes, and thus a key mediator of the type I IFN amplification loop [2], [9], [10]. Certain cells, including plasmacytoid dendritic cells and macrophages, express IRF-7 constitutively, which makes them poised for rapid IFN-α production [16]–[20].

West Nile virus (WNV) is a mosquito-transmitted, enveloped, positive-sense RNA virus and member of the *Flaviviridae* family. Studies in mice with targeted gene deletions have provided insight into mechanisms of innate immune restriction of WNV infection. The type I IFN response is essential to the control of WNV infection, as mice that are defective at producing or responding to IFN cannot control virus replication and succumb rapidly to infection [17], [21]–[25]. The host antiviral response *in vivo* is dependent upon both TLR and RLR signaling, as deficiencies in TLRs, RLRs, or their downstream adaptor molecules (including MyD88 and MAVS) result in enhanced viral replication and lethality [8], [22], [26]–[30].

Recent studies with WNV have suggested that some cell types use non-canonical signaling pathways to induce type I IFN responses. The combined absence of IRF-3 and IRF-7 resulted in uncontrolled WNV replication and more rapid death in *Irf3^−/−^×Irf7^−/−^* double knockout (DKO) mice compared to the individual single gene knockout mice [17], [21], [22], [31]. However, even without IRF-3 or IRF-7, type I IFN was produced by DKO mice infected with WNV or murine cytomegalovirus, albeit at reduced levels compared to wild type mice [22], [32]. Consistent with the sustained production of type I IFN, lethality in DKO mice infected with WNV or chikungunya virus was not as rapid or complete as in *Ifnar^−/−^* mice [22], [31], [33], [34]. *Ex vivo* experiments with primary myeloid dendritic cells (mDC) and macrophages revealed that the IFN-β response after WNV infection was sustained in DKO cells but abrogated in the absence of MAVS [22], [27]. In contrast, the IFN-β response in neurons and fibroblasts was abolished in the absence of either IRF-3 and IRF-7 or MAVS [22], [27]. These studies suggested cell type-specific requirements for the transcription factors that induce IFN-β expression in response to WNV infection.

To define the transcription factor(s) responsible for the IRF-3 and IRF-7-independent production of IFN-β in myeloid cells, we considered another member of the IRF family, IRF-5. Although IRF-5 was originally identified as an inducer of inflammatory cytokines (IL-6 and TNF-α) downstream of TLR-7 and MyD88 signaling, subsequent studies suggested that it could contribute to type I IFN production after viral infection [35]–[37]. In response to Newcastle disease virus (NDV) infection, IRF-5 induced overlapping and distinct sets of genes compared to IRF-7, including stronger induction of IFN-β and the antiviral gene *Rsad2* (Viperin) [38]. We generated *Irf3^−/−^*×*Irf5^−/−^*×*Irf7^−/−^* triple knockout (TKO) mice and found that these mice were highly vulnerable to infection with WNV. The combined loss of IRF-3, IRF-5, and IRF-7 largely abrogated type I IFN and ISG expression in mDC, and microarray analysis of WNV-infected mDC revealed a set of genes induced in DKO but not in TKO cells. Because the limited set of genes induced in WNV-infected TKO mDCs was absent in *Mavs^−/−^* mDCs, we conclude that the RLR-MAVS signaling pathway dominantly regulates innate immune gene induction in mDCs during WNV infection, and that IRF-3, IRF-5, and IRF-7 coordinately mediate this response. Our results establish a new linkage between the IRF-5 and the RLR signaling pathways in induction of the antiviral IFN response.

## Results

### TKO mice are highly vulnerable to viral infections

**Figure 1:**
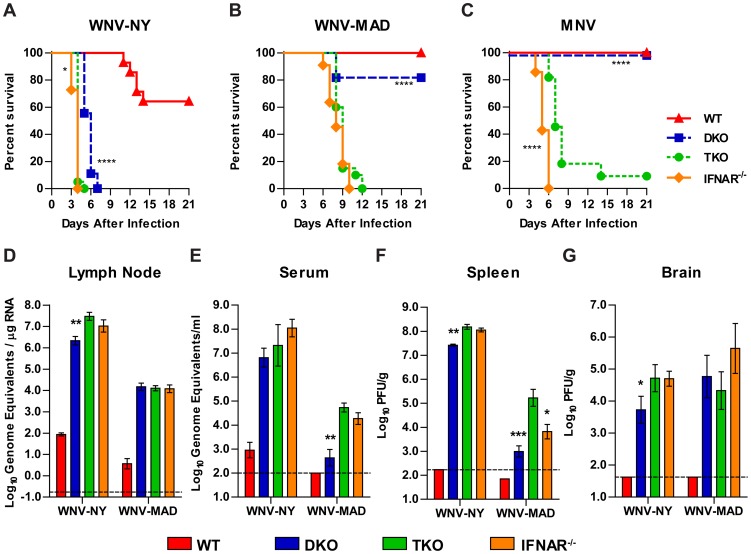
Lethality and viral burden after virus infection. **A–C**. WT, *Irf3^−/−^×Irf7^−/−^* DKO, *Irf3^−/−^×Irf5^−/−^×Irf7^−/−^* TKO, and *Ifnar^−/−^* mice were infected subcutaneously with 10^2^ PFU of a virulent (WNV-NY) or an attenuated (WNV-MAD) WNV strain or infected orally with 3×10^7^ PFU of MNV and followed for lethality for 21 days (8 to 20 mice per group). DKO and *Ifnar^−/−^* survival curves were compared to TKO by the log-rank test; asterisks indicate survival curves that are significantly different (****, *P*<0.0001; ***, *P*<0.001; *, *P*<0.05). **D–G**. The indicated groups of mice were infected subcutaneously with WNV-NY or WNV-MAD and tissues were harvested for viral burden analysis at 2 (WNV-NY) or 6 (WNV-MAD) days after infection. Viral infection in the draining lymph node and serum was determined by qRT-PCR, and infection in the spleen and brain was determined by plaque assay. Data are expressed as the mean viral titer ± standard error of the mean (SEM) of 5 to 9 mice per group and the dotted line represents the limit of detection of the assay. DKO and *Ifnar^−/−^* groups were compared to TKO by the Mann-Whitney test; asterisks indicate differences that are statistically significant (****, *P*<0.0001; ***, *P*<0.001; **, *P*<0.01; *, *P*<0.05).

We hypothesized that IRF-5 might be responsible for the residual IFN-β production in DKO mice, because IRF-5 contributes to *Ifnb* mRNA expression downstream of the PRR TLR7 and adaptor molecule MyD88, both of which limit WNV pathogenesis *in vivo* [28], [30], [39]. To test this, we generated *Irf3^−/−^×Irf5^−/−^×Irf7^−/−^* TKO mice (**Figure S1**) and defined their response to viral infection. TKO mice were viable, fertile, and produced progeny according to normal Mendelian frequencies (data not shown). We infected WT, DKO, and TKO mice with a virulent WNV strain (New York 2000, WNV-NY) and found that TKO mice succumbed to infection earlier than DKO mice (mean time to death (MTD): 4.0 days versus 5.7, *P*<0.0001). TKO mice died marginally later than *Ifnar^−/−^* mice, which do not respond to type I IFN and fail to control WNV replication (MTD: 4.0 days versus 3.7, *P*<0.05) (**Figure 1A**) [25], [31]. Because TKO, DKO and *Ifnar^−/−^* mice all succumbed so rapidly to WNV-NY infection, it was difficult to appreciate biologically meaningful differences in susceptibility among the three genotypes. To address this, we infected these mice with an attenuated WNV strain (Madagascar 1978, WNV-MAD) that inefficiently antagonizes JAK/STAT signaling [23]. With this virus, we observed a pronounced increase in mortality of TKO compared to DKO mice (**Figure 1B**). Whereas 100% of TKO mice succumbed to WNV-MAD infection, only 20% of DKO mice died (*P*<0.001). TKO mice were equally vulnerable to WNV-MAD infection as *Ifnar^−/−^* mice (*P*>0.05), and no statistical difference in MTD was observed (9.0 days for TKO versus 8.2 days for *Ifnar^−/−^* mice, *P*>0.05). Similar results were observed upon infection with murine norovirus (MNV), an unrelated non-enveloped positive-sense RNA virus. TKO mice were more vulnerable to MNV infection than DKO mice, with only 1 of 11 TKO mice surviving, compared to 100% survival for DKO mice (*P*<0.0001) (**Figure 1C**). However, the TKO mice did not show the same susceptibility as *Ifnar^−/−^* mice (*P*<0.0001), and the MTD was greater in TKO compared to *Ifnar^−/−^* mice (7.8 days versus 5.3 days, *P*<0.001). The observation that lethality in TKO mice more closely matched that of *Ifnar^−/−^* mice after WNV infection compared to MNV suggests that there may be virus-specific differences in the particular transcription factors responsible for mediating the antiviral response. Overall, the loss of IRF-5 in the setting of an IRF-3 and IRF-7 deficiency renders mice more vulnerable to viral infection and early death, approaching that seen in mice that cannot respond to type I IFN.

To understand the basis of the increased susceptibility of TKO mice to viral infection, we infected WT, DKO, TKO, and *Ifnar^−/−^* mice with WNV-NY or WNV-MAD and measured viral burden in the draining lymph node, serum, spleen and brain at 2 days (WNV-NY) or 6 days (WNV-MAD) after infection (**Figure 1D–G**). Viral infection in TKO mice was similar to that observed in *Ifnar^−/−^* mice (*P*>0.05) in all tissues examined, except for the spleen after WNV-MAD infection where titers in TKO mice were greater than in *Ifnar^−/−^* mice (25-fold, *P*<0.05). After infection with WNV-NY, TKO mice had higher viral loads than DKO mice in the draining lymph node (13-fold, *P*<0.01), spleen (5-fold, *P*<0.01), and brain (9-fold, *P*<0.05). After infection with WNV-MAD, TKO mice had higher viral loads than DKO mice in the serum (124-fold, *P*<0.01) and spleen (169-fold, *P*<0.01).

### Serum antiviral activity

**Figure 2:**
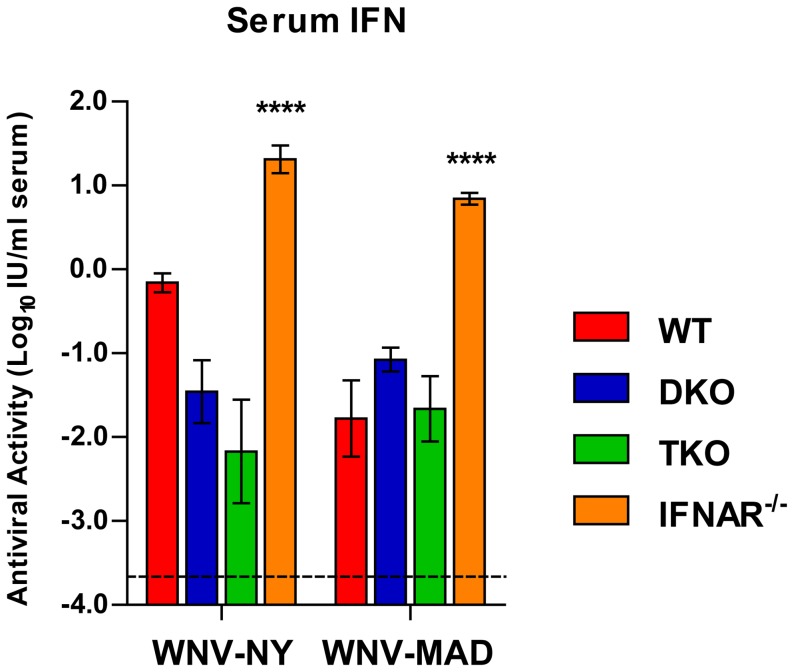
Type I IFN activity in serum. WT, *Irf3^−/−^×Irf7^−/−^* DKO, *Irf3^−/−^×Irf5^−/−^×Irf7^−/−^* TKO, and *Ifnar^−/−^* mice were infected with 10^2^ PFU of WNV-NY or WNV-MAD and serum levels of type I IFN were measured 2 (WNV-NY) or 6 (WNV-MAD) days after infection using a bioassay. Data represent the mean ± SEM of 5 to 9 mice per group. DKO and *Ifnar^−/−^* groups were compared to TKO by a two-way ANOVA; asterisks indicate differences that are statistically significant (****, *P*<0.0001).

To determine whether the enhanced vulnerability of TKO mice was due to an inability to generate a systemic antiviral response, we measured type I IFN levels in the serum of mice infected with WNV-NY (2 days after infection) or WNV-MAD (6 days after infection) (**Figure 2**). Unexpectedly, we detected type I IFN activity in the serum of TKO mice infected with WNV-NY or WNV-MAD, and the amount present was not different from DKO mice (*P*>0.05). While the serum levels of type I IFN in TKO and DKO mice were diminished compared to WT mice after WNV-NY infection and equivalent to WT after WNV-MAD infection, substantially higher levels of type I IFN were detected in the serum from *Ifnar^−/−^* mice (29-fold after WNV-NY infection, *P*<0.01; 416-fold after WNV-MAD infection, *P*<0.0001). The high level of type I IFN in *Ifnar^−/−^* mice likely is a result of high viral replication in the absence of IFN-mediated antiviral effector functions combined with the absence of IFNAR molecules to bind and internalize type I IFN in the serum. Despite the combined absence of IRF-3, IRF-5, and IRF-7, TKO mice still produced type I IFN after WNV infection, albeit at lower levels in the context of markedly enhanced infection.

### Virus control and ISG induction is ablated in TKO mDC but not macrophages

**Figure 3:**
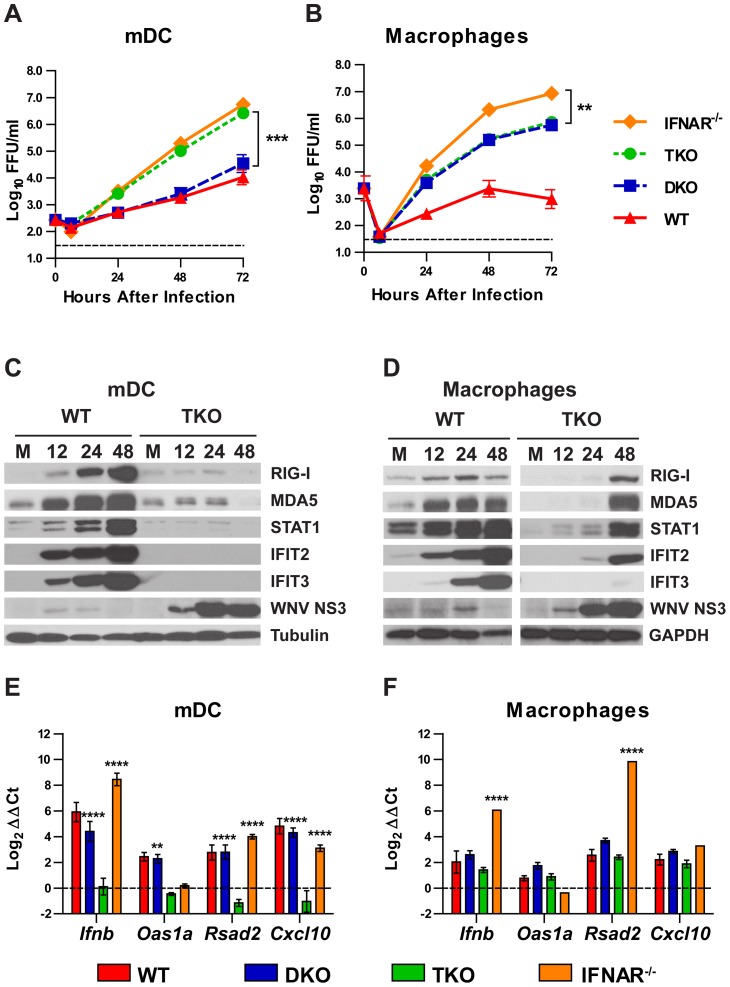
ISG and IFN-β induction in primary myeloid cells in response to WNV infection. Primary myeloid cells from WT, *Irf3^−/−^×Irf7^−/−^* DKO, *Irf3^−/−^×Irf5^−/−^×Irf7^−/−^* TKO, and *Ifnar^−/−^* mice were infected with WNV-NY. **A** and **B**. Bone marrow-derived mDC (**A**) and macrophages (**B**) were infected at an MOI of 0.001 (mDC) or 0.01 (macrophages), and viral replication was measured by focus-forming assay. Data represent the mean ± SEM of two independent experiments performed in triplicate. The dotted line represents the limit of detection of the assay. **C** and **D**: WT and TKO mDCs (**C**) and macrophages (**D**) were infected at an MOI of 1 or mock-infected (M). At 12, 24, or 48 hours after infection, cells were lysed, separated by SDS-PAGE and analyzed by western blot to detect expression of specific ISGs or viral proteins. One representative experiment of three is shown. **E** and **F:** mDCs (**E**) and macrophages (**F**) were infected with WNV at an MOI of 0.1, RNA was isolated at 24 hours after infection, and relative expression of the indicated target genes was measured by qRT-PCR. Gene expression was normalized to *Gapdh* and is displayed as the fold increase compared to uninfected cells on a log_2_ scale. Data represent the average of three independent experiments and are expressed as the mean ± SEM. **A**–**B** and **E**–**F**: DKO and *Ifnar^−/−^* groups were compared to TKO by two-way ANOVA; asterisks indicate differences that are statistically significant (****, *P*<0.0001; ***, *P*<0.001; **, *P*<0.01).

Myeloid cells retain the ability to produce IFN-β during WNV infection despite the lack of IRF-3 and IRF-7 [22]. To determine if this IFN-dependent antiviral activity was mediated by IRF-5, we performed multi-step growth analyses with WNV-NY in primary mDC and macrophages derived from WT, DKO, TKO, and *Ifnar^−/−^* mice (**Figure 3A and B**). Viral replication in TKO mDC was greater than in DKO mDC (74-fold, *P*<0.0001) and equivalent to *Ifnar^−/−^* mDC (*P*>0.05), suggesting that IRF-3, IRF-5, and IRF-7 regulate innate immune defense to control WNV replication in mDC. In comparison, TKO macrophages showed little increase in WNV-NY replication compared to DKO cells, and reached lower (11-fold, *P*<0.0001) peak titers compared to *Ifnar^−/−^* macrophages. This suggests that macrophages can restrict WNV-NY infection through an alternative pathway that is independent of IRF-3, IRF-5, and IRF-7, possibly through IRF-1 and/or other transcription factors [**40**].

To establish whether the disparate ability of TKO mDC and macrophages to control WNV-NY replication was associated with differences in antiviral gene induction, we infected cells and performed western blots to assay expression of ISGs, specifically RIG-I (DDX58), MDA5 (IFIH1), STAT1, IFIT2 (ISG54) and IFIT3 (ISG49) (**Figure 3C and D**). In TKO mDCs, we did not detect expression of any of the tested ISGs, although these were highly expressed in WNV-infected WT and DKO mDC (**Figure 3C** and [22]). In contrast, most of these proteins were induced in TKO macrophages, although their expression was delayed compared to WT cells: ISG expression was detected in TKO macrophages at only 48 hours after infection, whereas expression was detected in WT cells within 12 hours of infection. Unlike other ISGs, IFIT3 was not expressed in TKO macrophages even at 48 hours after infection, despite being induced in DKO macrophages [**22**]. The lack of virus-induced ISG expression in TKO mDC resembled the phenotype observed in cells lacking the RLR-signaling adaptor, MAVS [27].

To further define the ISGs expressed in an IRF-3, IRF-5, or IRF-7 dependent manner, we infected mDC and macrophages from WT, DKO, TKO, and *Ifnar^−/−^* mice with WNV-NY and measured the induction of *Ifnb*, *Oas1a*, *Rsad2*, and *Cxcl10* mRNA at 24 hours after infection by quantitative reverse transcription polymerase chain reaction (qRT-PCR) (**Figure 3E and F**). These genes were selected as known representatives of different ISG induction pathways. *Rsad2* and *Cxcl10* can be induced directly by PRR signaling and IRF-3 mediated transcriptional regulation, whereas expression of *Oas1a* depends more strictly upon IFN-β signaling [14], [15], [20]. Consistent with the western blot results, all four genes were induced strongly in WT and DKO mDC, but not in TKO mDC. In contrast, TKO macrophages retained the ability to express *Ifnb* and the tested ISGs, although the level of gene induction was equivalent to or less than WT cells, even in the context of enhanced viral replication. As expected, *Oas1a* was not induced in *Ifnar^−/−^* cells, although these cells expressed high levels of *Rsad2*, *Cxcl10* and *Ifnb*. ISG expression in *Ifnar^−/−^* macrophages was especially high, likely secondary to increased viral replication and IRF-3-dependent gene induction.

### TKO mDC respond to IFN-β treatment but not to PRR stimulation

**Figure 4:**
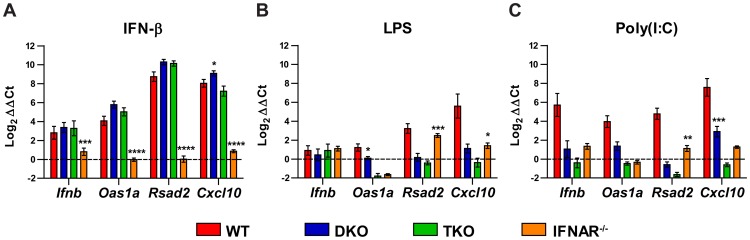
ISG induction by IFN-β and TLR agonists in WT and deficient mDC. mDC from WT, *Irf3^−/−^×Irf7^−/−^* DKO, *Irf3^−/−^×Irf5^−/−^×Irf7^−/−^* TKO, and *Ifnar^−/−^* mice were treated with IFN-β (500 IU/ml) (**A**), poly(I∶C) (50 µg/ml) (**B**), or LPS (5 µg/ml) (**C**). Total RNA was isolated 24 hours later and relative gene expression was measured by qRT-PCR. Gene expression was normalized to *Gapdh* and is displayed as the fold increase compared to untreated cells on a log_2_ scale. Data represent the average of three independent experiments and are expressed as the mean ± SEM. DKO and *Ifnar^−/−^* groups were compared to TKO by two-way ANOVA; asterisks indicate differences that are statistically significant (****, *P*<0.0001; ***, *P*<0.001; **, *P*<0.01; *, *P*<0.05).

Since TKO mDC failed to induce expression of selected ISGs in response to WNV-NY infection, we tested their capacity to express ISGs in response to other inflammatory stimuli including IFN-β and the PRR agonists poly(I∶C) and lipopolysaccharide (LPS) (**Figure 4**). Although TKO mDC failed to induce *Ifnb* expression after WNV-NY infection, they retained the ability to respond to its signaling, inducing WT levels of *Ifnb*, *Oas1a*, *Rsad2*, *and Cxcl10* at 24 hours after IFN-β treatment. However, these cells showed an ablated response to poly(I∶C) or LPS, with no induction of *Ifnb* or the tested ISGs. Thus, TKO mDC are defective in transmitting MyD88- and TRIF-dependent signals after PAMP sensing, whereas the JAK/STAT-ISGF3 signaling pathway remains intact. As observed previously, although DKO mDC induced a WT-like pattern of ISGs after WNV infection, they had a diminished response to stimulation by the TLR4 ligand LPS or by poly(I∶C), which is recognized by TLR3 and MDA5 [22]. This suggests that WNV infection activates a broader range of PRRs than poly(I∶C) or LPS treatment alone.

### Microarray analysis reveals a MAVS-dependent signal through IRF-5 in mDCs

**Figure 5:**
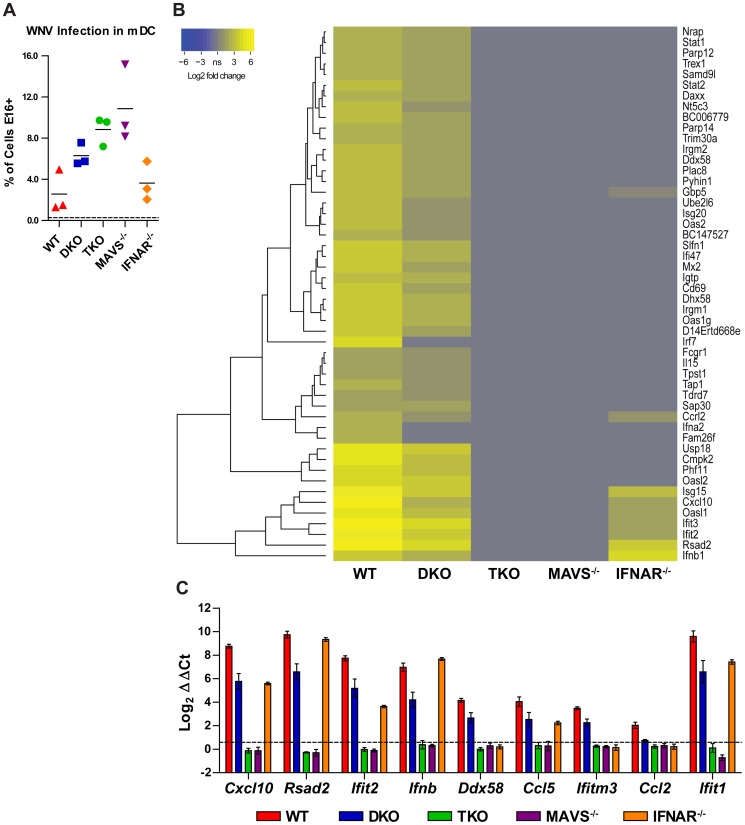
Microarray analysis of WNV infected mDC. mDC from WT, *Irf3^−/−^×Irf7^−/−^* DKO, *Irf3^−/−^×Irf5^−/−^×Irf7^−/−^* TKO, *Mavs^−/−^* and *Ifnar^−/−^* mice were infected with WNV-NY at an MOI of 25 and total RNA was harvested 24 hours later. **A.** WNV infection of mDC from the indicated genotypes as assessed by anti-WNV MAb staining at 24 hours after infection. **B**. Heatmap showing the 50 genes with the greatest fold change in expression in WNV-infected mDC compared to mock-infected cells, according to the indicated color scale. The gray portion of the color scale, labeled “ns” for non-significant, represents genes that failed to meet the cutoff criteria for induction. Gene expression was assessed by microarray analysis on Illumina chips. Each column represents the mean of three independent samples per genotype. **C.** Quantitative RT-PCR was performed on the same RNA samples analyzed by microarray to detect expression of the indicated target genes. Gene expression was normalized to *Gapdh* and is displayed as the fold increase compared to mock-infected cells on a log_2_ scale. Data represent the average of three independent samples and are expressed as the mean ± SEM. The dotted line indicates a 1.5-fold increase in expression.

Analysis of selected ISGs in TKO mDC infected with WNV-NY suggested a profound loss of gene induction, results that also were seen previously in *Mavs^−/−^* cells [27]. To evaluate this in greater detail, we performed a microarray analysis to profile gene expression patterns in TKO and *Mavs^−/−^* mDC 24 hours after WNV-NY infection at a multiplicity of infection (MOI) of 25. To identify the specific contributions of IRF-5 and type I IFN signaling to the transcriptional response, studies also were performed with WT, DKO, and *Ifnar^−^* ^/−^ mDCs. The level of WNV infection of the cells used for the microarray was assessed by flow cytometry using an anti-WNV monoclonal antibody (**Figure 5A**). TKO and *Mavs^−/−^* mDC had significantly higher rates of infection compared to WT cells (*P*<0.05 and *P*<0.01, respectively), whereas infection of DKO and *Ifnar^−/−^* mDC surprisingly was not different than WT (*P*>0.05). Nonetheless, for all genotypes tested, only a fraction (up to 15%) of cells stained positive for WNV antigen at 24 hours after infection, suggesting that uninfected cells contributed substantially to the gene induction profile observed in this experiment.

Gene induction was measured by comparing WNV-infected samples to mock-infected cells of the same genotype, to control for differential basal expression of some genes. We considered genes to be expressed differentially in response to WNV infection if they exhibited a fold change of ≥1.5 and a *P*-value<0.05. WNV-infected WT mDCs showed a broad transcriptional response, particularly of genes that are induced by PRR and type I IFN signaling. 445 genes were expressed differentially in WNV-infected mDC compared to mock-infected cells (**Table S1**). The 50 most upregulated genes (**Figure 5B**) included ISGs with previously described antiviral activity (*Rsad2*, *Ifit2*, *Ifit3*, *Isg15*, *Isg20*, and *Parp12*) [13], [41]–[43], members of the 2′-5′-oligoadenylate synthetase family (*Oas1g*, *Oas2*, *Oasl1*, and *Oasl2*) [12], [44], [45], components of the PRR/type I IFN (*Ddx58*, *Dhx58*, *Ifnb1*, *Ifna2*, *Irf7*, *Stat1*, and *Stat2*) and ISG15 (*Isg15*, *Ube2l6*, *Usp8*) [12] pathways, as well as nucleotide metabolism factors (*Cmpk2* and *Nt5c3*). The particular genes upregulated in DKO mDC were similar to those in WT cells, although the magnitude of induction was lower in DKO cells, consistent with previous observations [22]. In contrast, a restricted set of 22 genes was expressed differentially in WNV-infected *Ifnar^−^* ^/−^ mDCs (**Figure 5B** and **Table S2**). Remarkably few genes were expressed differentially in either TKO or *Mavs^−^* ^/−^ mDC upon WNV-NY infection, suggesting that the RLR signaling pathway is critical for initiating the type I IFN and antiviral responses in this cell type.

To validate the results of the microarray analysis, we performed qRT-PCR with the same RNA samples that were used for transcriptional profiling (**Figure 5C**) and measured the expression of *Cxcl10*, *Rsad2*, *Ifit2*, *Ifnb*, *Ddx58*, *Ccl5*, *Ifitm3*, and *Ccl2*. The induction pattern measured by qRT-PCR corroborated the microarray results. These eight genes (listed above in order of relative expression level) were induced in WT and DKO cells but not in TKO or *Mavs^−/−^* cells. Consistent with the patterns observed by microarray, *Cxcl10*, *Rsad2*, *Ifit2*, *Ifnb*, and *Ccl5* were induced in *Ifnar^−/−^* cells (i.e., are IFN-independent), whereas *Ddx58*, *Ifitm3*, and *Ccl2* were not (i.e., are IFN-dependent). *Ifit1* (ISG56) is an ISG that is highly upregulated upon WNV infection [17], [21], [27], [46]–[49], thus its absence from the infection-induced bioset was unexpected. Upon further analysis by qRT-PCR, we found that *Ifit1* was induced to high levels in infected WT, DKO, and *Ifnar^−/−^* mDC but not TKO or *Mavs^−/−^* cells. This quality control assessment reveals that the single *Ifit1* probe on our microarray chip was defective, and that *Ifit1* expression is induced in *Ifnar^−^* ^/−^ cells after WNV infection.

**Figure 6:**
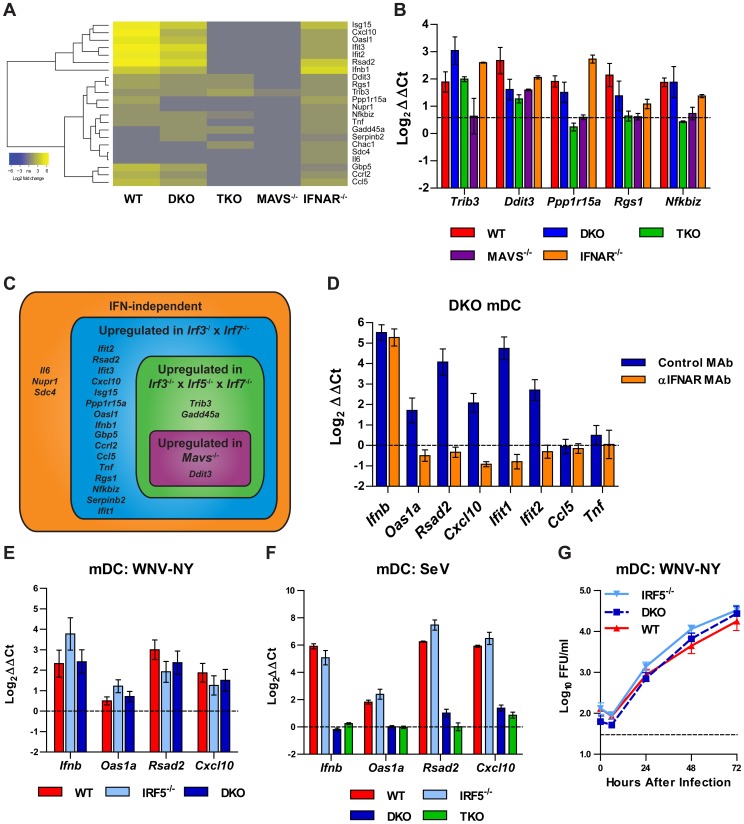
Type I IFN signaling mediates gene induction by IRF-5 and MAVS. **A.** Heatmap displaying 22 genes induced upon WNV infection in *Ifnar^−/−^* mDC (greater than 1.5 fold upregulated compared to mock-infected cells, *P*<0.05). **B.** Expression of selected IFN-independent genes was validated by qRT-PCR using the same RNA samples analyzed by microarray. Gene expression was normalized to *Gapdh* and is displayed as the fold increase compared to mock-infected cells on a log_2_ scale. Data represent the average of three independent samples and are expressed as the mean ± SEM. The dotted line indicates a 1.5-fold increase in expression. **C.** Venn diagram of the expression patterns of IFN-independent genes, based on microarray and qRT-PCR analyses. **D.** DKO mDC were treated with 25 µg/ml of an IFNAR-blocking antibody (MAR1-5A3) or an isotype control antibody (GIR-208) for one hour prior to infection with WNV-NY at an MOI of 0.1. Total RNA was isolated after 24 hours and relative gene expression was measured by qRT-PCR. Expression of the indicated target genes was normalized to *Gapdh* and is displayed as the fold increase compared to untreated cells on a log_2_ scale. Data represent the average of four samples from two independent experiments and are expressed as the mean ± SEM. **E.** mDC from WT, *Irf5^−/−^*, and DKO mice were infected with WNV-NY at an MOI of 0.1 and qRT-PCR was performed as in panel **D**. Data represent the average of 12 samples from four independent experiments, are displayed as the fold increase compared to untreated cells on a log_2_ scale, and are expressed as the mean ± SEM. **F.** mDC from WT, *Irf5^−/−^*, DKO, and TKO mice were infected with SeV at an MOI of 3 and qRT-PCR was performed as in panel **D**. Data represent the average of six samples from two independent experiments, are displayed as the fold increase compared to untreated cells on a log_2_ scale, and are expressed as the mean ± SEM. **G.** mDC from WT and *Irf5^−/−^* mice were infected at an MOI of 0.001 and viral replication was measured by focus-forming assay. Data represent the mean ± SEM of six independent experiments performed in triplicate. WT and *Irf5^−/−^* titers were compared by two-way ANOVA and were not significantly different (*P*>0.05). The dotted line represents the limit of detection of the assay.

To identify genes whose expression was dependent strictly upon IRF-5 and MAVS, we considered those upregulated in WT but not in *Mavs^−/−^* cells (MAVS-dependent) or in WT and DKO but not in TKO cells (IRF-5 dependent). Since TKO and *Mavs^−/−^* mDC failed to produce IFN-β in response to WNV infection (**Figure 3** and [27]), we stratified our analysis to consider only genes that were upregulated in *Ifnar^−/−^* mDC, so as to exclude those whose differential expression might be secondary to the lack of IFN signaling in *Mavs^−/−^* and TKO cells. The IFN-independent set of genes (**Figure 6A** and **Table S2 and S3**) included *Ifnb1*, *Rsad2*, *Isg15*, *Cxcl10*, *Ifit2*, and *Ifit3*, all of which are induced by IRF-3 without a requirement for IFNAR-mediated signaling [14], [15]. Further analysis revealed that IFN-independent genes included cytokines (*Ifnb1*, *Tnf*, *Il6*), chemokines (*Cxcl10*, *Ccl5*, *Ccrl2*), antiviral restriction factors (*Rsad2*, *Isg15*, *Ifit2*, *Ifit3*), and components of the unfolded protein response (*Ppp1r15a* (GADD34), *Ddit3* (CHOP, GADD153), *Chac1*). To corroborate this analysis, we measured the expression of *Trib3*, *Ddit3*, *Ppp1r15a*, *Rgs1*, *Nfkbiz*, and *Chac1* by qRT-PCR using the same RNA samples used for the microarray (**Figure 6B**). We confirmed that three of these genes were upregulated in WNV-infected TKO mDC (*Trib3*, *Ddit3*, and *Gadd45a*) (**Figure 6C**). The qRT-PCR data did however, yield some differences: (a) *Trib3* induction was not detected in *Mavs^−/−^* mDC by qRT-PCR; (b) *Ddit3* was upregulated in a MAVS-independent manner; (c) *Rgs1* and *Nfkbiz* were not upregulated in TKO cells; (d) while *Ppp1r15a* was upregulated in *Ifnar^−/−^* mDC, it also was induced in DKO mDC; and (e) by qRT-PCR we failed to detect expression of *Chac1* in mock- or WNV-infected mDC of any genotype, although it was induced in WNV-infected cortical neurons (data not shown).

The absence of gene induction in TKO mDC compared to DKO cells could reflect a direct role for IRF-5 in ISG induction or an indirect effect of the loss of IFN-β production in TKO mDC. To test this, we inhibited type I IFN signaling in DKO cells using an IFNAR-blocking monoclonal antibody (MAR1-5A3, [50]) and used qRT-PCR to measure gene induction in response to WNV-NY infection (**Figure 6D**). As expected, the IFNAR-blocking antibody prevented induction of *Oas1a*, a known IFN-dependent ISG [15], but did not impair induction of *Ifnb*. *Ccl5* and *Tnf* were induced too weakly to observe differences between the IFNAR-blocking and control MAbs. However, the IFNAR-blocking antibody abolished induction of *Cxcl10*, *Rsad2*, *Ifit1*, and *Ifit2*, even though these genes are considered to be IFN-independent [14], [15] and were induced in *Ifnar^−/−^* mDC (**Figure 5C**). Collectively, these results suggest that IRF-5 contributes to the induction of IFN-β expression after WNV infection in mDC, but does not induce ISG expression directly. To further define the contribution of IRF-5 to IFN and ISG induction in mDC, we infected WT, *Irf5^−/−^*, and DKO mDC with WNV (**Figure 6E**) and WT, *Irf5^−/−^*, DKO, and TKO cells with Sendai virus (SeV), a negative sense RNA paramyxovirus (**Figure 6F**) and measured gene expression by qRT-PCR. We found no change in the induction of *Ifnb*, *Oas1a*, *Rsad2*, or *Cxcl10* in *Irf5^−/−^* mDC compared to WT cells (*P*>0.05), indicating that loss of IRF-5 alone in mDCs is not sufficient to impact the antiviral response, analogous to results seen with IRF-3 [21]. Consistent with this observation, we observed no significant difference in WNV-NY replication between *Irf5^−/−^* and WT mDC (*P*>0.05) (**Figure 6G**). Although DKO mDC retained intact IFN and ISG responses after WNV infection, this pattern surprisingly was not observed following SeV infection: the induced expression of several ISGs (*Oas1a*, *Rsad2*, and *Cxcl10*) was lost in both DKO and TKO mDC. While our results with DKO and TKO cells after WNV infection establish that IRF-5 contributes to the type I IFN response in mDCs, the critical nature of the IFN induction pathways in these key sentinel cells may have resulted in the maintenance of redundant signaling pathways to sustain antiviral gene programs. Indeed, the distinct ISG induction phenotypes after WNV and SeV infection in DKO and TKO mDCs suggest that activation of these parallel pathways may differ among diverse viruses.

**Figure 7:**
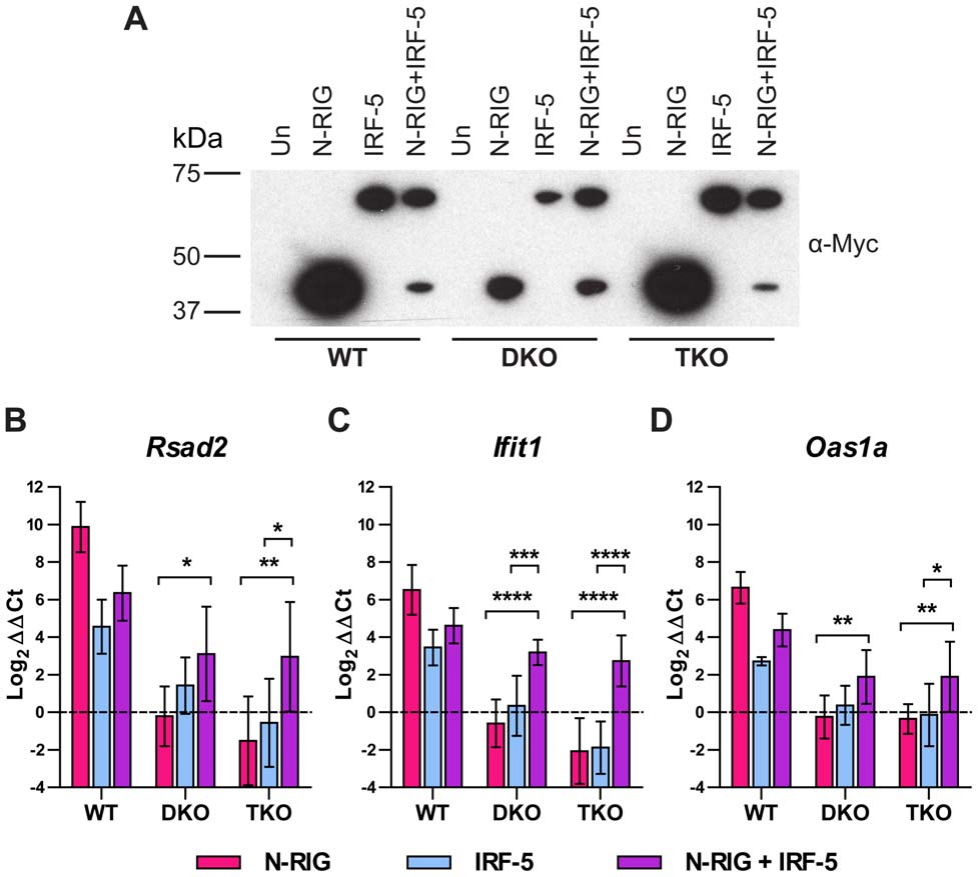
WT, *Irf3^−/−^×Irf7^−/−^* DKO, and *Irf3^−/−^×Irf5^−/−^×Irf7^−/−^* TKO immortalized MEFs were transfected with plasmids expressing myc-tagged IRF-5 or residues 1–229 of RIG-I (N-RIG) and analyzed at 24 hours after transfection by western blot (A) or qRT-PCR (B–D). **A.** Transfected cell lysates were separated by SDS-PAGE and N-RIG or IRF-5 were detected with an anti-myc-tag antibody. Un: no transfection. Expression of N-RIG and IRF-5 was decreased slightly upon co-transfection, likely secondary to promoter competition. **B–D.** Expression of the indicated ISGs was measured from total RNA by qRT-PCR. Gene expression was normalized to *Gapdh* and is displayed as the fold increase compared to untransfected cells on a log_2_ scale. Data represent the average of four samples from two independent experiments and are expressed as the mean ± SEM. The co-transfection group was compared to transfection with the individual plasmids by two-way ANOVA for DKO and TKO groups; asterisks indicate differences that are statistically significant (****, *P*<0.0001; ***, *P*<0.001; **, *P*<0.01; *, *P*<0.05).

The similar gene induction profiles observed between TKO and *Mavs^−/−^* mDC by microarray and qRT-PCR suggested a functional interaction between IRF-5 and MAVS. To test this hypothesis, we transfected WT, DKO, and TKO immortalized mouse embryonic fibroblasts (MEFs) with plasmids encoding myc-tagged forms of a constitutively active RIG-I (N-RIG) and/or IRF-5. Ectopic expression of N-RIG and IRF-5 was detected in MEFs 24 hours after transfection by western blotting (**Figure 7A**) and qRT-PCR (data not shown). As expected, we observed increased expression of ISGs (e.g., *Rsad2*, *Ifit1*, and *Oas1a*) in WT MEFs transfected with N-RIG compared to untransfected cells (**Figure 7B–D**). Transfection of N-RIG alone in DKO cells failed to induce these ISGs, suggesting that endogenous IRF-5 in MEFs is not adequately expressed or activated to induce ISGs after a MAVS-dependent signal; these results agree with prior studies showing that the combined loss of IRF-3 and IRF-7 in MEFs abolished the ISG response after WNV infection [22], [27]. In comparison, co-transfection of N-RIG and IRF-5 together but not IRF-5 alone enhanced ISG induction in DKO and TKO MEFs. Thus, MAVS-dependent induction of ISGs can occur through an IRF-5-dependent yet IRF-3 and IRF-7-independent pathway.

## Discussion

In the present study, we generated *Irf3^−/−^*×*Irf5^−/−^*×*Irf7^−/−^* TKO mice to establish that these three IRF family transcription factors coordinately regulate IFN-β production and ISG expression in mDC. We found that antiviral gene induction was ablated almost entirely in mDC from TKO or *Mavs^−/−^* mice, suggesting a dominant role for MAVS in initiating the antiviral response and pointing to a novel signaling interaction between IRF-5 and the RLR signaling pathway.

As TKO mice succumbed to WNV infection with similar kinetics compared to *Ifnar^−/−^* mice, we expected they would be completely defective at producing type I IFN. Nonetheless, we detected type I IFN activity in the serum of infected TKO mice, suggesting that some cells must produce type I IFN by a pathway that is independent of IRF-3, IRF-5, and IRF-7. Macrophages or related cells (e.g., inflammatory monocytes) may be one source of this residual type I IFN *in vivo*, as TKO macrophages cultured *ex vivo* expressed *Ifnb* as well as a subset of ISGs in response to WNV infection. Type I IFN induction in TKO macrophages could be mediated in part by IRF-1, which regulates expression of antiviral genes independently of type I IFN in the context of several other viral infections [13], [51], [52]. Consistent with this, *Irf1^−/−^* macrophages supported enhanced WNV replication compared to WT controls [40], and viral replication in TKO macrophages did not phenocopy *Ifnar^−^* ^/−^ cells. Nonetheless, IRF-1 was not sufficient to induce the full complement of ISGs in macrophages, as *Ifnb* and ISG expression in TKO macrophages was diminished and delayed compared to WT cells. Furthermore, IFIT3 was not expressed in TKO macrophages, although it was sustained in DKO cells [22]. It remains unclear whether the genes upregulated in TKO macrophages were induced by IRF-1 directly, by another transcription factor, or downstream of IFN-β production by these cells.

We measured ISG induction in infected mDC to determine whether a lack of antiviral effector gene expression explained the failure of TKO mice and mDC to control WNV replication. In our experiments, fewer than 15% of mDC were infected at 24 hours, even when a high MOI of 25 was used. Increasing the MOI to 100 achieved only marginally higher rates of infection (data not shown) and was not practical for the scale of the microarray experiments. Sorting infected cells by flow cytometry prior to transcriptional profiling analysis was not feasible as infected cells must be permeabilized to detect intracellular WNV antigens and recombinant WNV expressing green fluorescent protein are attenuated and/or unstable [53]–[55]. In our microarray studies, uninfected cells likely contributed substantially to the ISG expression signatures observed. Indeed, few genes were induced in WNV-infected TKO or *Mavs^−/−^* mDC, even though these cells would be expected to upregulate genes associated with cell stress, survival, and metabolism in response to replication by a cytopathic virus. Some components of the unfolded protein response, including *Ddit3* and *Gadd45a*, were upregulated in infected TKO mDC; additional genes likely were induced in infected cells but may have been below the statistical cutoffs used in our analysis due to dilution of the transcripts in a large pool of mRNA from uninfected cells.

Viral infection induces the expression of ISGs both directly (by IRF-3 after PAMP detection and PRR signaling) and indirectly (by IFN-β production and IFNAR signaling), the latter occurring in both infected and uninfected cells. Given the large proportion of uninfected cells, we would expect genes induced by IFNAR signaling to predominate. Indeed, only a small subset of genes was induced after WNV infection of *Ifnar^−/−^* mDC (22 genes, compared to 445 in WT mDC). This may reflect the relatively low infection rates, an inherent inefficiency of IFNAR-independent gene induction pathways, or viral countermeasures that antagonize the type I IFN response in highly infected cells [56]. Of the 22 genes induced in WNV-infected *Ifnar^−/−^* mDC, several (*Ifnb*, *Cxcl10*, *Rsad2*, *Ifit1*, and *Ifit2*) have direct or indirect antiviral activity against WNV [13], [24], [41], [42], [57]–[59] and are induced directly by IRF-3 [14], [15]. Other genes induced in WNV-infected *Ifnar^−/−^* mDC included components of the unfolded protein response, such as *Ddit3* and *Ppp1r15a*. *Ddit3* (CHOP) has been shown to promote expression of *Ppp1r15a* (*Gadd34*) and *Trib3* [60]–[62], two IFN-independent induced genes detected in our microarray analysis. While induction of these genes may represent a response to the cellular stress caused by viral infection, the unfolded protein response also constitutes a cellular defense that limits replication of diverse viruses, including WNV [60], [63], [64]. DDIT3 inhibits WNV replication, and WNV may induce expression of *Ppp1r15a* to reverse DDIT3-mediated translational inhibition [60]. In contrast, PPP1R15A is required for IFN-β production and contributes to controlling replication of chikungunya virus [65].

Although global gene induction in response to WNV infection has been reported previously [46]–[49], [66], [67], our results represent the first such analysis in DCs, which are a sentinel cell type coordinating the innate and adaptive antiviral immune responses, as well as among the first cells infected following a mosquito bite [8], [68]. Some of the genes we identified in mDCs also were detected in microarray analyses of WNV-infected MEFs [46], human kidney epithelial cells [48], or human retinal pigmented epithelium [47]. Induction of these genes (e.g., *Rsad2*, *Ifit2*, *Isg15*, *Isg20*, and *Stat1*) thus does not depend on cell type-specific transcription factors. Other WNV-induced genes, however, may be specific to DCs or restricted cell types. As an example, the chemokine *Cxcl10* was one of the most highly induced genes in our analysis, yet it was induced at much lower levels or not at all in fibroblasts and epithelial cells [46]–[48]. CXCL10 contributes to clearance of WNV infection from the CNS by recruiting effector T cells, and is the dominant chemokine secreted by neurons after WNV infection [57].

Only one of the 22 genes differentially expressed in *Ifnar^−/−^* mDC, *Ddit3*, was induced in *Mavs^−/−^* mDC, suggesting that the IFN-independent induction signal is conveyed almost entirely by MAVS. Since *Mavs^−/−^* mDC failed to produce IFN-β, we surmise that both type I IFN-dependent and -independent pathways of ISG induction are abrogated in these cells. This conclusion agrees with earlier studies on induction of selected sets of genes in *Mavs^−/−^* mDC infected with WNV or rabies virus [27], [69]. Although *Mavs^−/−^* cells should retain TLR-mediated antiviral gene induction pathways (which signal through TRIF and MyD88), we observed almost no ISG induction in *Mavs^−/−^* mDC after WNV infection. Thus, RLRs likely are the dominant PRRs that sense WNV infection in mDC; these results are consistent with the essentially intact antiviral responses reported in WNV-infected *Tlr3^−/−^* and *Myd88^−/−^* mDC [26], [28].

**Figure 8:**
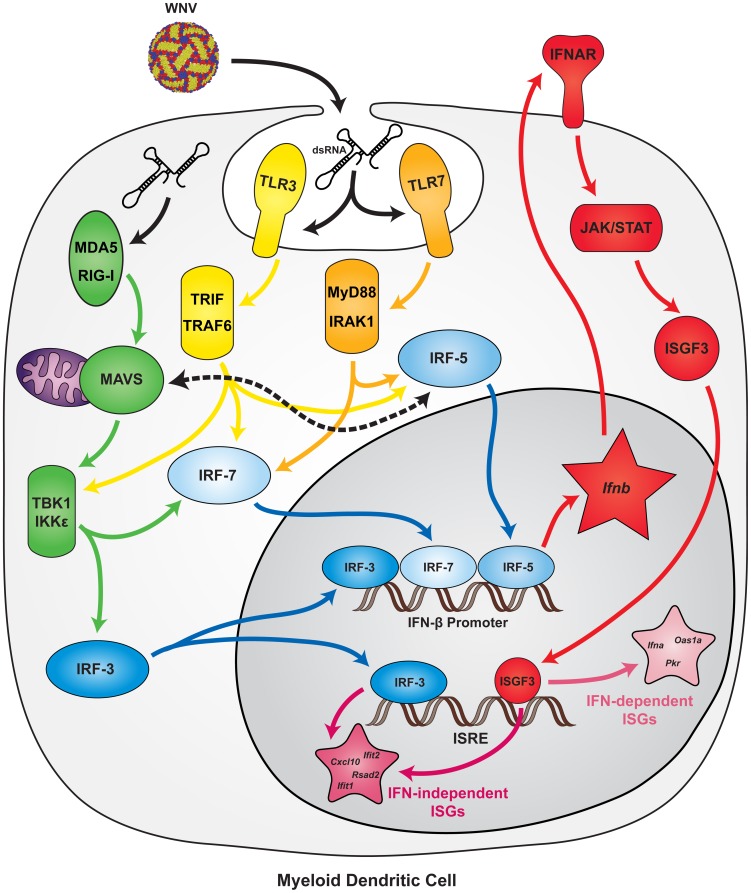
Model of Type I IFN and ISG induction in mDC. WNV infection is sensed by PRR from the RLR family (RIG-I and MDA5, *green*) or TLR family (TLR3 and TLR7, *yellow and orange*). PRR signal through their respective adaptor molecules (MAVS, TRIF, MyD88), which activates cellular kinases (TBK1, IKKε, TRAF6, IRAK1). Phosphorylation of IRF-3, IRF-5, and IRF-7 (*blue*) induces nuclear localization, and in concert with other transcription factors (e.g., NF-κB), results in induced expression of *Ifnb* and ISGs. IRF-3, IRF-5, and IRF-7 are each sufficient to induce expression of IFN-β (*red*), which can signal through IFNAR to activate expression of hundreds of ISGs (*pink*). Some ISGs, including *Ifna*, *Oas1a*, and *Pkr*, are dependent strictly upon IFN signaling for their induction. Others, including *Ifit1*, *Ifit2*, *Rsad2*, and *Cxcl10*, can be induced directly by IRF-3, although IRF-5 apparently is not sufficient to induce these genes independently of IFN signaling. In addition to being activated by TLR7 signaling through MyD88, IRF-5 is activated by MAVS through an uncharacterized pathway.

Although our microarray and qRT-PCR analyses identified 16 genes that were differentially expressed in WNV-infected *Ifnar^−/−^* and DKO but not TKO mDC, when gene expression was analyzed from WNV-infected DKO cells that were treated with an antibody blocking type I IFN signaling, only *Ifnb* gene induction was sustained. These data suggest that in the absence of IRF-3 and IRF-7, IRF-5 is sufficient to induce IFN-β production in response to WNV infection, but unlike IRF-3 [14], [15], does not induce ISGs directly (**Figure 8**). Although IRF-5 has been suggested to promote IFN-independent expression of some ISGs including *Pkr* and *Isg20* in NDV-infected cells [38], IRF-3 may have contributed to these responses. The observed anti-WNV response in DKO mDC likely results from IRF-5-dependent IFN-β production, and the uncontrolled viral replication in TKO mDC is secondary to a lack of IFN-β and resultant absence of ISG induction. This model suggests that cell types having ancillary pathways for IFN-β induction (such as IRF-1 in macrophages) can mount antiviral responses even in the absence of IRF-3, IRF-5, and IRF-7.

We did not anticipate that the *Mavs^−^* ^/−^ and TKO mDC would phenocopy one other with respect to ISG induction, since IRF-5 has not been previously implicated in the RLR signaling pathway [35]–[37]. IRF-5 originally was described as an inducer of pro-inflammatory cytokines (e.g., IL-6 and TNF-α) but subsequently was suggested to contribute to the type I IFN antiviral response. *Irf5^−/−^* mice have increased susceptibility to viral infections, slightly reduced levels of type I IFN in serum, and more significantly reduced levels of pro-inflammatory cytokines [35], [37]. IRF-5 expression and antiviral activity, however, appears restricted to a limited set of cell types, including monocytes and DCs [35], [39], [70]. Thus, a relative absence of IRF-5 expression in fibroblasts and neurons may explain the observation that type I IFN induction after WNV infection in these cell types is abolished by the combined deletion of IRF-3 and IRF-7 [22]. However, the ability of alternate IRFs to compensate for IRF-3 and IRF-7 in fibroblasts also may depend on the particular viral stimulus, as type I IFN production was essentially absent in DKO fibroblasts infected with WNV, herpes simplex virus, vesicular stomatitis virus, or encephalomyocarditis virus [19], [22], but low-level production of *Ifnb* and *Ifna2* mRNA was sustained in DKO fibroblasts infected with chikungunya virus [33]. IRF-5 preferentially stimulates the IFN-β and IFN-α4 promoters, rather than other IFN-α subtypes, which also suggests that it contributes to the primary type I IFN response, prior to amplification via autocrine and paracrine signaling [35]. The IFN-α subtypes induced in IRF-5-expressing cells vary from those induced in IRF-7-expressing cells, suggesting that the IRF expression patterns within a cell modulate the breadth of the type I IFN response [70].

Although MAVS previously was known to induce IFN-β production via IRF-3 and IRF-7, our experiments suggest that RLR signaling also activates IRF-5 to induce IFN-β production in mDC; the subcellular location where this occurs (e.g., mitochondrion) and through what signaling intermediates remains unknown. A recent study suggested that activation of RLR signaling acts to inhibit induction of inflammatory cytokines by IRF-5 [71]; although the net result was different, this study is consistent with our observation of a functional interaction between IRF-5 and MAVS and with a prior proteomic study demonstrating a physical interaction between these two proteins [72]. Future studies will be required to delineate the mechanistic and functional intermediates that link and regulate the IRF-5 and RLR signaling pathways.

## Materials and Methods

### Viruses

The WNV-NY strain (3000.0259) was isolated in New York in 2000 and passaged once in C6/36 *Aedes albopictus* cells to generate a virus stock that was used in all experiments except for the microarray analysis [73], [74]. For the microarray studies, mDCs were infected in the Früh laboratory with the WNV New York 1999 strain that was propagated in C6/36 cells [75]. The attenuated strain WNV-MAD was amplified in Vero cells and has been previously described [23]. MNV strain MNV1.CW3 [76] was propagated in RAW 264.7 cells (ATCC) and a concentrated stock was prepared as previously described [77]. The SeV virus strain Fushimi was propagated in chicken embryos and provided by D. Lenschow and M. Holtzman (Washington University, St Louis, MO).

### Ethics statement

This study was carried out in strict accordance with the recommendations in the Guide for the Care and Use of Laboratory Animals of the National Institutes of Health. The protocol was approved by the Institutional Animal Care and Use Committee at the Washington University School of Medicine (Assurance Number: A3381-01). Dissections and footpad injections were performed under anesthesia that was induced and maintained with ketamine hydrochloride and xylazine, and all efforts were made to minimize suffering.

### Mouse experiments

All mice used were on an inbred C57BL/6 background. WT mice were commercially obtained (Jackson Laboratories). *Irf3^−/−^×Irf7^−/−^* DKO, *Irf5^−/−^*, and *Ifnar^−/−^* mice have been reported previously [22], [31], [36]. *Irf3^−/−^×Irf5^−/−^×Irf7^−/−^* TKO mice were generated by crossing DKO and *Irf5^−/−^* mice. *Irf5^−/−^* and TKO mice were genotyped for a mutation in the *Dock2* gene, which can arise spontaneously in some *Irf5^−^* ^/−^ mice [78]; none of the TKO mice had homozygous mutations in *Dock2*. *Mavs^−/−^* mice were generated directly from C57BL/6 embryonic stem cells [34]. All deficient mice were bred in the animal facilities of the Washington University School of Medicine and genotyped prior to experimentation. For WNV infections, 10^2^ PFU was diluted in Hank's Balanced Salt Solution supplemented with 1% heat-inactivated fetal bovine serum and 8 to 12 week-old mice were inoculated by footpad injection in a volume of 50 µl. For MNV infections, 7 to 8 week-old mice were inoculated orally with 3×10^7^ PFU in 25 µl of PBS and monitored for survival for 21 days.

### Measurement of viral burden

To monitor viral spread *in vivo*, mice were infected with 10^2^ PFU of virus and sacrificed at 2 days after infection (WNV-NY) or 6 days after infection (WNV-MAD). After extensive perfusion with PBS, organs were harvested, weighed, homogenized and virus was titered by plaque assay on BHK21-15 cells [74]. Viral burden in serum and inguinal lymph node was measured using fluorogenic qRT-PCR using primers and probes to WNV-NY or WNV-MAD envelope gene sequences (**Table S4**). Viral RNA in the lymph node was normalized to *Gapdh* levels in tissue samples. Viral RNA from serum was isolated using a Viral RNA Mini Kit (Qiagen). Total RNA from lymph nodes was extracted using the E.Z.N.A. total RNA kit (Omega Bio-tek) and DNase-treated to remove genomic DNA. Quantitative RT-PCR was performed using One-Step RT-PCR Master Mix and a 7500 Fast Real-Time PCR System (Applied Biosystems).

### Quantification of type I IFN activity

Levels of biologically active type I IFN in serum were determined using an encephalomyocarditis virus L929 cytopathic effect bioassay as described [79]. The amount of type I IFN per ml of serum was calculated from a standard curve using IFN-β (PBL InterferonSource) and adjusted for the background inhibitory activity of naïve serum (approximately 0.1 IU/ml). The inhibitory activity of naïve serum was type I IFN-independent because it was acid labile but resistant to treatment with heat (56°C) or the IFNAR-blocking antibody MAR1-5A3 [17], [50].

### Primary cell infections

Macrophage and mDC cultures were generated as described previously [79]. Briefly, bone marrow was isolated from WT, DKO, TKO, *Irf5^−/−^*, or *Ifnar^−/−^* mice and cultured for seven days in the presence of 40 ng/ml M-CSF (PeproTech) to generate macrophages or with 20 ng/ml GM-CSF and 20 ng/ml IL-4 (PeproTech) to produce mDC. Multi-step virus growth analysis was performed after infection at a MOI of 0.01 for macrophages or 0.001 for mDCs. Supernatants were titered by focus-forming assay on Vero cells using humanized E16 anti-WNV MAb as the detection antibody [80], horseradish peroxidase conjugated anti-human IgG (Sigma), and True Blue Peroxidase Substrate (KPL). For western blotting, cells were infected at an MOI of 1. For measurement of ISG induction by qRT-PCR, cells were infected at an MOI of 0.1. To block signaling by type I IFN, DKO cells were treated with 25 µg/ml of the IFNAR-blocking MAb MAR1-5A3 for one hour prior to infection. A non-binding MAb against human IFN-γ receptor (GiR-208) was used as an isotype control [50].

### Microarray analysis of mDCs

Bone marrow cells were cultured in RPMI supplemented with 10% fetal bovine serum, penicillin/streptomycin, L-glutamine, non-essential amino acids, 55 µM β-mercaptoethanol and 20 ng/ml recombinant mouse GM-CSF (eBioscience) for six days in non-tissue culture treated plates. GM-CSF was replenished after two days and non-adherent cells were sub-cultured after 4 days. Sub-cultured cells were infected at an MOI of 25 with WNV-NY. Total RNA was harvested at 0, 6, 12, and 24 hours post-infection with an RNeasy Mini Kit (Qiagen). RNA was treated with DNase prior to cDNA generation. Gene expression was assayed on Illumina microarray chips. Microarray datasets were processed by quantile normalization and annotated using the *illuminaMousev2.db* R package version 1.10.0. Data were assessed by linear modeling with the *limma* package [81]. Differentially expressed genes were identified as those with at least a 1.5-fold change as compared to controls and a *P*-value<0.05 without correction for false discovery. WNV-infected samples were first compared with mock-infected controls. Microarray data have been deposited in GeoArchive, series number GSE42232.

### Transfection and ectopic expression

MEFs prepared from WT, DKO, or TKO mice were immortalized after transfection with the plasmid pSV2, which encodes for the large T antigen of SV40. MEFs were transfected using Lipofectamine 2000 (Invitrogen) with plasmids expressing myc-tagged forms of murine IRF-5 (Origene) or residues 1–229 of human RIG-I (N-RIG) [82]. Cells were lysed 24 hours post-transfection and analyzed by qRT-PCR and western blotting.

### Western blotting

Macrophages and mDC were lysed in RIPA buffer (10 mM Tris, 150 mM NaCl, 0.02% sodium azide, 1% sodium deoxycholate, 1% Triton X-100, 0.1% SDS, pH 7.4), with protease inhibitors (Sigma). Samples (20 µg) were resolved by electrophoresis on 10% SDS-polyacrylamide gels. MEFs were lysed in RIPA buffer and lysates were separated by electrophoresis on 4–12% SDS-polyacrylamide gels. Following transfer of proteins, membranes were blocked with 5% non-fat dried milk and probed with the following panel of primary antibodies: rabbit anti-IFIT2 and -IFIT3 (provided by Dr. G. Sen, [83]); rabbit anti-RIG-I and anti-MDA5 (IBL); mouse anti-tubulin (Sigma); rabbit anti-GAPDH (Santa Cruz); rabbit anti-STAT1 (Cell Signaling); goat-anti WNV NS3 (R&D Systems); mouse anti-myc (Santa Cruz). Western blots were incubated with peroxidase-conjugated secondary antibodies (Jackson Immunoresearch and Sigma) and visualized using ECL reagents (Amersham Biosciences and Pierce).

### Measurement of ISG expression by qRT-PCR

mDCs were treated for 24 hours with 500 IU/ml of IFN-β (PBL Interferon Source), 50 µg/ml of poly(I∶C) (InvivoGen), or 5 µg/ml of LPS (List Biological Laboratories). Macrophages and mDC were infected with WNV-NY at an MOI 0.1 for 24 hours. MEFs were harvested 24 hours after transfection. Total RNA was extracted using the E.Z.N.A. total RNA kit (Omega Bio-tek) or RNeasy kit (Qiagen) and treated with DNase. Fluorogenic qRT-PCR was performed using One-Step RT-PCR Master Mix and a 7500 Fast Real-Time PCR System (Applied Biosystems) with the indicated Taqman primers and probes (**Table S4**). Gene induction was normalized to *Gapdh* levels and expressed on a log_2_ scale as fold increase over mock according to the ΔΔCt method [84].

### Statistical analysis

Data were analyzed with GraphPad Prism software. Viral burdens were compared using the Mann-Whitney test. Serum type I IFN levels, viral growth curves and qRT-PCR were compared using a 2-way ANOVA. Kaplan-Meier survival curves were analyzed by the log rank test and mean times to death were compared by Student's T-test.

## Supporting Information

Figure S1 **Genotyping of TKO mice.** DNA from the tails of the indicated mice was amplified by PCR using primers specific for IRF-3, IRF-5, or IRF-7 and separated by agarose gel electrophoresis. The band sizes confirmed the genotypes of the knockout mice.
(TIF)

Table S1 **Gene induction in WNV-NY infected mDC.** All genes (445) for which expression level in at least one genotype was ≥1.5-fold changed at 24 hours after WNV infection (*P*<0.05, without correction for false discovery). Values represent the mean of three independent samples for each genotype. “Fold change” refers to the relative fold change of expression in WNV-infected mDC compared with mock-infected controls of the same genotype. DKO: *Irf3^−/−^*×*Irf7^−/−^*; TKO: *Irf3^−/−^*×*Irf 5^−/−^*×*Irf7^−/−^*.
(DOCX)

Table S2 **IFN-independent gene induction.** Genes are shown for which expression level in *Ifnar^−/−^* mDC was ≥1.5-fold changed at 24 hours after WNV infection (*P*<0.05, without correction for false discovery). Values represent the mean of three independent samples for each genotype. “Fold change” refers to the relative fold change of expression in WNV-infected mDC compared with mock-infected controls of the same genotype. DKO: *Irf3^−/−^*×*Irf7^−/−^*; TKO: *Irf3^−/−^*×*Irf 5^−/−^*×*Irf7^−/−^*.
(DOCX)

Table S3 **Genes induced in IFNAR and DKO, but not TKO mDC.** Genes are shown for which expression level in *Ifnar^−/−^* and DKO mDC was ≥1.5-fold changed at 24 hours after WNV infection (*P*<0.05), but which fell short of these cutoffs in TKO cells. Values represent the mean of three independent samples for each genotype. “Fold change” refers to the relative fold change of expression in WNV-infected mDC compared with mock-infected controls of the same genotype. DKO: *Irf3^−/−^*×*Irf7^−/−^*; TKO: *Irf3^−/−^*×*Irf 5^−/−^*×*Irf7^−/−^*.
(DOCX)

Table S4 **Primers and probes used for quantitative RT-PCR.**
(DOCX)
