# Correction: IRF-3, IRF-5, and IRF-7 Coordinately Regulate the Type I IFN Response in Myeloid Dendritic Cells Downstream of MAVS Signaling

**DOI:** 10.1371/annotation/4de7ddfd-52df-4f87-8ca4-d48afe646ca8

**Published:** 2013-05-03

**Authors:** Helen M. Lazear, Alissa Lancaster, Courtney Wilkins, Mehul S. Suthar, Albert Huang, Sarah C. Vick, Lisa Clepper, Larissa Thackray, Margaret M. Brassil, Herbert W. Virgin, Janko Nikolich-Zugich, Ashlee V. Moses, Michael Gale, Klaus Früh, Michael S. Diamond

The GeoArchive accession number for the microarray data, GSE42232, is incorrect. It should instead be GSE41355.

