# Supplementary figures and images for: IRF-3, IRF-5, and IRF-7 Coordinately Regulate the Type I IFN Response in Myeloid Dendritic Cells Downstream of MAVS Signaling
Source: PLoS Pathog. 2013 Jan 3;9(1):e1003118. doi: 10.1371/journal.ppat.1003118 (PMC3536698; doi:10.1371/journal.ppat.1003118)

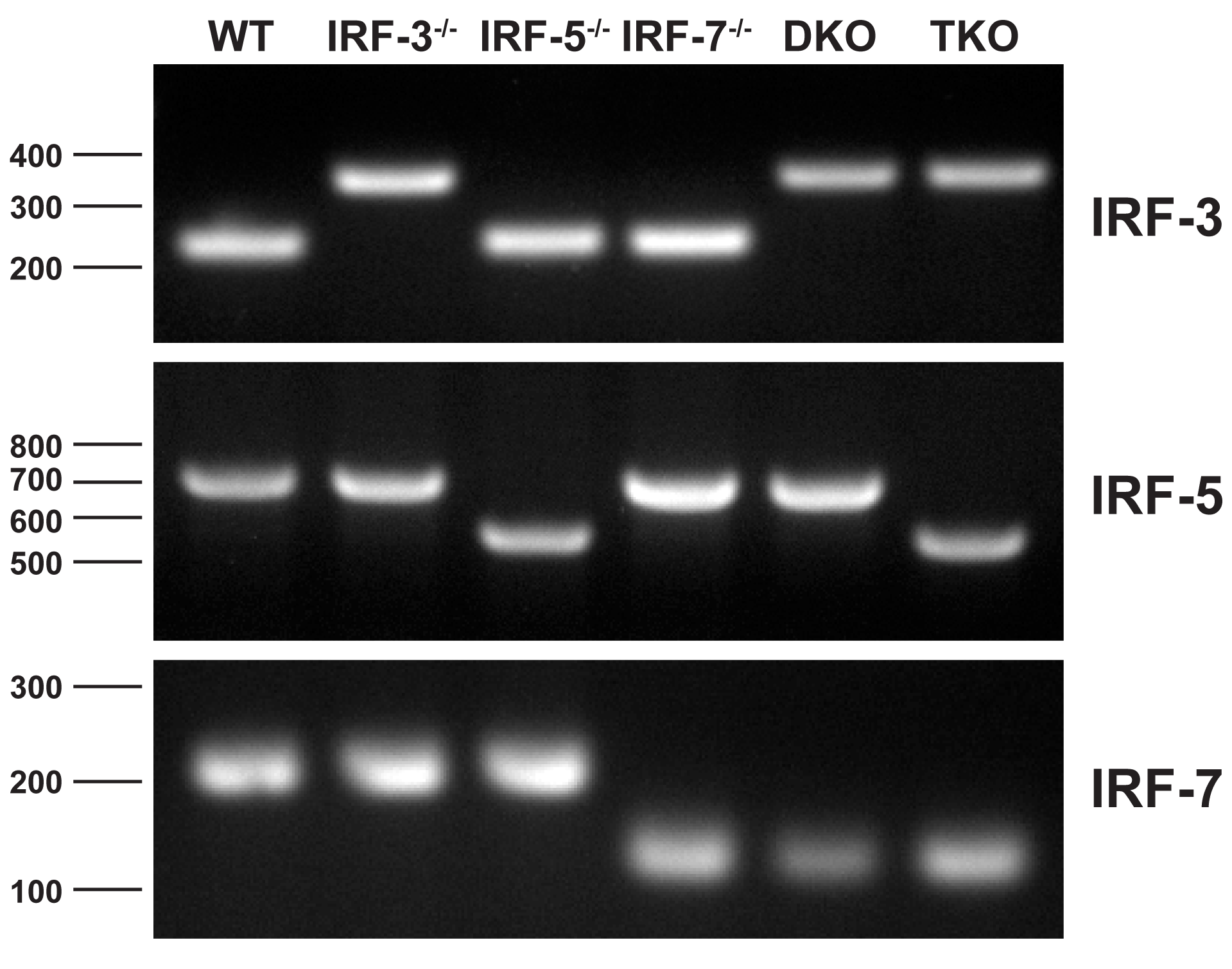

Supplement: Figure S1 — Genotyping of TKO mice. DNA from the tails of the indicated mice was amplified by PCR using primers specific for IRF-3, IRF-5, or IRF-7 and separated by agarose gel electrophoresis. The band sizes confirmed the genotypes of the knockout mice. (TIF) [file ppat.1003118.s001.tif]
