# Supplementary material for: IRF-3, IRF-5, and IRF-7 Coordinately Regulate the Type I IFN Response in Myeloid Dendritic Cells Downstream of MAVS Signaling
Source: PLoS Pathog. 2013 Jan 3;9(1):e1003118. doi: 10.1371/journal.ppat.1003118 (PMC3536698; doi:10.1371/journal.ppat.1003118)
Supplement: Table S1 — Gene induction in WNV-NY infected mDC. All genes (445) for which expression level in at least one genotype was ≥1.5-fold changed at 24 hours after WNV infection (P<0.05, without correction for false discovery). Values represent the mean of three independent samples for each genotype. “Fold change” refers to the relative fold change of expression in WNV-infected mDC compared with mock-infected controls of the same genotype. DKO: Irf3−/−×Irf7−/−; TKO: Irf3−/−×Irf 5−/−×Irf7−/−. (DOCX) [file ppat.1003118.s002.docx]

**Table S1: Gene induction in WNV-NY99 infected mDC**

|  | **WT** | | **DKO** | | **TKO** | | ***Mavs^-/-^*** | | ***Ifnar^-/-^*** | |
| --- | --- | --- | --- | --- | --- | --- | --- | --- | --- | --- |
| **Gene** | **Fold**  **change** | **P Value** | **Fold**  **change** | **P Value** | **Fold**  **change** | **P Value** | **Fold**  **change** | **P Value** | **Fold**  **change** | **P Value** |
| **Cxcl10** | 73.15 | 1.13E-26 | 6.03 | 2.42E-16 | 1.05 | 5.46E-01 | 1.02 | 8.37E-01 | 3.73 | 2.48E-14 |
| **Cmpk2** | 32.51 | 7.95E-24 | 8.38 | 1.78E-17 | 1.03 | 7.45E-01 | -1.07 | 4.57E-01 | 1.20 | 4.87E-02 |
| **Rsad2** | 99.45 | 2.26E-23 | 20.01 | 1.06E-17 | 1.03 | 8.41E-01 | -1.27 | 6.58E-02 | 11.90 | 6.82E-17 |
| **Ifit3** | 96.42 | 5.73E-23 | 17.49 | 6.58E-17 | 1.03 | 8.09E-01 | 1.27 | 7.27E-02 | 2.99 | 6.35E-09 |
| **Ifit2** | 55.22 | 7.44E-23 | 15.77 | 9.33E-18 | 1.00 | 9.95E-01 | -1.01 | 9.44E-01 | 3.00 | 5.82E-10 |
| **Phf11** | 23.80 | 4.06E-22 | 6.62 | 1.35E-15 | 1.11 | 2.69E-01 | 1.00 | 9.80E-01 | 1.12 | 2.43E-01 |
| **Ifi47** | 11.72 | 1.01E-21 | 4.20 | 5.40E-15 | 1.11 | 1.93E-01 | -1.13 | 1.37E-01 | 1.01 | 9.14E-01 |
| **Isg15** | 58.60 | 1.27E-20 | 11.85 | 2.28E-14 | 1.00 | 9.93E-01 | -1.04 | 7.86E-01 | 6.34 | 1.27E-12 |
| **Parp12** | 5.52 | 1.54E-20 | 2.80 | 3.18E-14 | 1.06 | 3.34E-01 | 1.02 | 7.35E-01 | 1.07 | 2.89E-01 |
| **Irf7** | 24.00 | 3.60E-20 | 1.05 | 7.26E-01 | -1.03 | 7.80E-01 | 1.03 | 8.24E-01 | -1.06 | 6.47E-01 |
| **Mx2** | 11.65 | 4.71E-20 | 3.43 | 5.04E-12 | 1.08 | 4.10E-01 | -1.02 | 8.21E-01 | 1.44 | 4.56E-04 |
| **D14Ertd668e** | 14.79 | 9.05E-20 | 2.83 | 1.88E-09 | 1.04 | 6.94E-01 | 1.03 | 7.96E-01 | 1.02 | 8.47E-01 |
| **Ifnb1** | 13.43 | 9.01E-18 | 4.29 | 5.04E-11 | 1.03 | 8.05E-01 | 1.05 | 6.87E-01 | 19.47 | 3.71E-19 |
| **Irgm1** | 10.02 | 4.74E-19 | 4.92 | 3.58E-14 | -1.09 | 3.55E-01 | -1.03 | 7.24E-01 | 1.06 | 5.19E-01 |
| **Oasl1** | 27.11 | 6.23E-19 | 7.72 | 5.42E-13 | -1.03 | 8.18E-01 | 1.04 | 7.62E-01 | 3.42 | 2.06E-09 |
| **BC147527** | 5.96 | 2.46E-18 | 2.22 | 1.66E-09 | 1.08 | 3.40E-01 | 1.00 | 9.52E-01 | 1.01 | 9.46E-01 |
| **Ube2l6** | 7.00 | 3.21E-18 | 1.95 | 2.55E-07 | -1.03 | 7.38E-01 | 1.07 | 4.32E-01 | 1.11 | 2.47E-01 |
| **Daxx** | 6.18 | 3.82E-18 | 2.73 | 3.46E-11 | -1.09 | 3.01E-01 | -1.06 | 4.45E-01 | -1.01 | 8.55E-01 |
| **Usp18** | 29.66 | 5.28E-18 | 10.99 | 2.10E-13 | -1.02 | 9.12E-01 | 1.06 | 6.82E-01 | -1.02 | 9.08E-01 |
| **Nt5c3** | 6.50 | 1.02E-17 | 2.46 | 1.27E-09 | -1.02 | 7.91E-01 | 1.03 | 7.33E-01 | 1.08 | 3.74E-01 |
| **Irgm2** | 7.89 | 1.44E-17 | 3.52 | 1.35E-11 | -1.05 | 6.39E-01 | 1.05 | 5.85E-01 | -1.06 | 5.40E-01 |
| **Oas1g** | 11.03 | 1.54E-17 | 5.63 | 3.80E-13 | -1.13 | 2.89E-01 | -1.01 | 9.40E-01 | 1.05 | 6.72E-01 |
| **Pyhin1** | 7.66 | 2.05E-17 | 2.94 | 3.29E-10 | -1.05 | 6.18E-01 | 1.02 | 8.51E-01 | 1.23 | 3.97E-02 |
| **Stat2** | 6.53 | 2.11E-17 | 2.76 | 2.24E-10 | -1.00 | 9.84E-01 | 1.08 | 4.19E-01 | 1.06 | 5.12E-01 |
| **Ddx58** | 7.26 | 4.49E-17 | 3.59 | 1.12E-11 | -1.05 | 6.19E-01 | 1.07 | 5.06E-01 | 1.00 | 9.80E-01 |
| **Dhx58** | 10.68 | 4.54E-17 | 5.19 | 2.23E-12 | -1.18 | 1.69E-01 | -1.00 | 9.97E-01 | -1.03 | 7.85E-01 |
| **Cd69** | 10.25 | 5.30E-17 | 3.78 | 1.63E-10 | 1.01 | 9.19E-01 | 1.10 | 4.02E-01 | 1.38 | 9.03E-03 |
| **Ifna2** | 5.32 | 1.23E-16 | -1.01 | 9.42E-01 | 1.05 | 5.93E-01 | 1.10 | 2.81E-01 | 1.46 | 1.53E-04 |
| **Tpst1** | 3.53 | 1.24E-16 | 1.88 | 4.80E-09 | -1.01 | 9.01E-01 | 1.08 | 2.27E-01 | -1.07 | 3.21E-01 |
| **Fcgr1** | 3.90 | 1.37E-16 | 1.76 | 1.46E-07 | 1.02 | 7.35E-01 | -1.11 | 1.57E-01 | -1.04 | 5.63E-01 |
| **Fam26f** | 4.01 | 1.52E-16 | 1.46 | 7.24E-05 | 1.02 | 7.91E-01 | -1.01 | 9.38E-01 | 1.27 | 2.54E-03 |
| **Il15** | 3.72 | 1.91E-16 | 1.73 | 1.61E-07 | 1.03 | 6.85E-01 | 1.00 | 9.96E-01 | 1.00 | 9.60E-01 |
| **Parp14** | 6.03 | 2.62E-16 | 3.75 | 3.32E-12 | -1.11 | 2.77E-01 | -1.13 | 2.14E-01 | 1.02 | 8.32E-01 |
| **Samd9l** | 4.65 | 2.79E-16 | 2.69 | 5.75E-11 | -1.21 | 2.72E-02 | -1.02 | 7.83E-01 | 1.15 | 8.95E-02 |
| **Slfn1** | 12.92 | 3.40E-16 | 4.45 | 5.26E-10 | -1.08 | 5.56E-01 | 1.02 | 8.97E-01 | -1.03 | 8.11E-01 |
| **Isg20** | 6.35 | 4.52E-16 | 1.95 | 2.93E-06 | -1.08 | 4.75E-01 | -1.03 | 7.42E-01 | -1.01 | 9.37E-01 |
| **Tap1** | 4.14 | 5.46E-16 | 2.00 | 2.29E-08 | -1.03 | 7.28E-01 | 1.05 | 5.20E-01 | 1.03 | 7.30E-01 |
| **Oasl2** | 22.97 | 5.75E-16 | 15.09 | 1.95E-13 | -1.11 | 5.52E-01 | 1.02 | 9.24E-01 | 1.46 | 3.62E-02 |
| **Gbp5** | 7.34 | 1.01E-15 | 2.79 | 1.39E-08 | 1.02 | 8.30E-01 | 1.08 | 4.77E-01 | 1.51 | 1.11E-03 |
| **Oas2** | 7.04 | 1.20E-15 | 2.22 | 8.37E-07 | -1.05 | 6.85E-01 | -1.01 | 9.23E-01 | -1.03 | 7.84E-01 |
| **Plac8** | 8.47 | 1.32E-15 | 2.77 | 7.23E-08 | 1.16 | 2.20E-01 | -1.13 | 3.15E-01 | 1.08 | 5.10E-01 |
| **Tdrd7** | 2.97 | 1.73E-15 | 1.67 | 9.85E-08 | -1.09 | 1.68E-01 | 1.01 | 9.21E-01 | 1.08 | 2.36E-01 |
| **Trex1** | 4.83 | 1.75E-15 | 2.61 | 9.03E-10 | 1.04 | 6.41E-01 | -1.14 | 1.53E-01 | 1.07 | 4.38E-01 |
| **BC006779** | 6.39 | 2.35E-15 | 3.07 | 1.35E-09 | 1.09 | 4.43E-01 | 1.12 | 3.13E-01 | 1.08 | 4.90E-01 |
| **Nod1** | 2.97 | 3.14E-15 | 1.41 | 6.95E-05 | 1.04 | 5.54E-01 | -1.00 | 9.55E-01 | -1.10 | 1.56E-01 |
| **Nrap** | 5.27 | 4.89E-15 | 2.74 | 2.34E-09 | -1.15 | 1.71E-01 | 1.07 | 4.81E-01 | 1.20 | 7.79E-02 |
| **Oas1b** | 2.51 | 6.55E-15 | 1.45 | 3.21E-06 | 1.07 | 2.59E-01 | -1.03 | 5.67E-01 | 1.16 | 1.53E-02 |
| **Stat1** | 5.05 | 7.47E-15 | 2.69 | 2.88E-09 | -1.09 | 4.07E-01 | 1.06 | 5.86E-01 | 1.00 | 9.95E-01 |
| **Enpp4** | 6.02 | 1.09E-14 | 1.52 | 2.44E-03 | -1.13 | 2.91E-01 | 1.16 | 1.93E-01 | 1.03 | 8.23E-01 |
| **Cxcl9** | 3.46 | 1.27E-14 | 1.20 | 4.69E-02 | 1.08 | 3.49E-01 | 1.05 | 4.99E-01 | 1.12 | 1.66E-01 |
| **Ccrl2** | 4.56 | 1.51E-14 | 2.14 | 1.91E-07 | 1.14 | 1.74E-01 | -1.04 | 6.87E-01 | 1.93 | 3.12E-07 |
| **Fcgr4** | 6.38 | 1.65E-14 | 1.53 | 3.24E-03 | -1.08 | 5.38E-01 | -1.14 | 2.66E-01 | 1.01 | 9.23E-01 |
| **Adar** | 2.60 | 1.71E-14 | 1.68 | 4.91E-08 | -1.13 | 4.72E-02 | 1.07 | 2.50E-01 | -1.13 | 5.56E-02 |
| **Prm1** | 2.91 | 1.80E-14 | 1.11 | 1.73E-01 | -1.02 | 7.93E-01 | -1.01 | 9.19E-01 | 1.02 | 8.26E-01 |
| **Ifi204** | 5.71 | 2.13E-14 | 2.76 | 1.68E-08 | 1.09 | 4.26E-01 | -1.00 | 9.70E-01 | 1.05 | 6.92E-01 |
| **Igtp** | 9.41 | 2.30E-14 | 4.12 | 4.02E-09 | -1.09 | 5.44E-01 | -1.03 | 8.49E-01 | -1.01 | 9.21E-01 |
| **AA960436** | 2.68 | 2.61E-14 | 1.22 | 1.09E-02 | 1.07 | 3.27E-01 | 1.09 | 2.10E-01 | 1.02 | 7.51E-01 |
| **Tor3a** | 3.54 | 2.88E-14 | 1.97 | 9.15E-08 | 1.08 | 3.42E-01 | 1.02 | 8.25E-01 | 1.07 | 4.06E-01 |
| **Oas3** | 7.51 | 3.15E-14 | 2.75 | 3.22E-07 | 1.03 | 8.42E-01 | 1.02 | 8.99E-01 | -1.12 | 3.83E-01 |
| **Trim30a** | 5.11 | 3.92E-14 | 3.40 | 2.15E-10 | 1.05 | 6.36E-01 | 1.21 | 9.00E-02 | -1.02 | 8.66E-01 |
| **Uba7** | 5.80 | 4.42E-14 | 1.96 | 2.38E-05 | 1.04 | 7.33E-01 | 1.00 | 9.76E-01 | -1.00 | 9.92E-01 |
| **Pnp** | 3.75 | 5.32E-14 | 1.71 | 1.11E-05 | -1.03 | 7.39E-01 | 1.10 | 2.72E-01 | -1.03 | 7.14E-01 |
| **Gm12597** | 3.81 | 5.86E-14 | -1.12 | 2.83E-01 | 1.03 | 7.48E-01 | -1.11 | 2.45E-01 | 1.22 | 3.66E-02 |
| **Sap30** | 3.52 | 6.77E-14 | 2.55 | 4.16E-10 | -1.08 | 3.71E-01 | -1.06 | 5.04E-01 | 1.08 | 3.61E-01 |
| **Batf2** | 3.72 | 8.36E-14 | 1.58 | 1.10E-04 | 1.05 | 5.95E-01 | -1.05 | 5.80E-01 | -1.12 | 2.18E-01 |
| **Gbp3** | 5.98 | 9.58E-14 | 2.27 | 2.82E-06 | 1.28 | 5.62E-02 | -1.07 | 6.07E-01 | 1.21 | 1.37E-01 |
| **Trim30d** | 4.83 | 1.73E-13 | 3.54 | 2.10E-10 | -1.06 | 5.97E-01 | 1.14 | 2.60E-01 | 1.02 | 8.61E-01 |
| **Serpina3f** | 2.45 | 1.98E-13 | 1.20 | 1.44E-02 | 1.07 | 3.04E-01 | 1.02 | 7.99E-01 | 1.05 | 4.13E-01 |
| **Sp100** | 4.54 | 2.53E-13 | 2.50 | 6.00E-08 | -1.02 | 8.80E-01 | 1.08 | 4.92E-01 | 1.01 | 9.24E-01 |
| **Rilpl1** | 4.60 | 2.96E-13 | 1.02 | 8.89E-01 | 1.01 | 8.98E-01 | 1.04 | 7.39E-01 | -1.03 | 8.14E-01 |
| **Epsti1** | 2.45 | 3.08E-13 | 1.61 | 7.16E-07 | 1.11 | 1.31E-01 | -1.03 | 7.04E-01 | -1.06 | 3.52E-01 |
| **Ly6a** | 17.92 | 3.15E-13 | 3.24 | 3.24E-05 | -1.21 | 3.67E-01 | -1.07 | 7.62E-01 | -1.23 | 3.34E-01 |
| **Lgals3bp** | 3.97 | 3.23E-13 | 2.61 | 5.74E-09 | -1.30 | 1.50E-02 | 1.26 | 2.72E-02 | -1.08 | 4.57E-01 |
| **Ms4a6d** | 3.34 | 3.46E-13 | 1.41 | 1.59E-03 | -1.08 | 3.95E-01 | 1.09 | 3.57E-01 | -1.06 | 5.30E-01 |
| **Ifna14** | 3.44 | 5.79E-13 | -1.05 | 6.42E-01 | 1.10 | 3.23E-01 | -1.03 | 7.46E-01 | 1.06 | 5.60E-01 |
| **Fabp3** | 5.09 | 5.94E-13 | 1.02 | 8.76E-01 | 1.05 | 7.15E-01 | -1.05 | 7.12E-01 | 1.01 | 9.29E-01 |
| **Eif2ak2** | 4.27 | 6.76E-13 | 3.39 | 2.76E-10 | -1.25 | 5.01E-02 | 1.02 | 8.39E-01 | -1.02 | 8.89E-01 |
| **Irf9** | 3.07 | 9.11E-13 | 2.41 | 1.46E-09 | -1.01 | 9.49E-01 | 1.29 | 5.89E-03 | -1.01 | 9.03E-01 |
| **Ifi27l2a** | 6.12 | 1.24E-12 | 2.59 | 2.16E-06 | 1.02 | 9.02E-01 | -1.19 | 2.29E-01 | 1.10 | 5.08E-01 |
| **Ogfr** | 3.05 | 1.26E-12 | 1.53 | 1.55E-04 | 1.05 | 5.50E-01 | -1.15 | 1.16E-01 | 1.12 | 2.07E-01 |
| **Cd40** | 6.76 | 1.26E-12 | 2.76 | 1.79E-06 | 1.15 | 3.47E-01 | 1.08 | 5.92E-01 | 1.35 | 5.24E-02 |
| **H2-T22** | 4.54 | 1.49E-12 | 1.69 | 5.04E-04 | 1.26 | 5.88E-02 | 1.13 | 3.01E-01 | 1.07 | 5.94E-01 |
| **Irf1** | 3.84 | 1.69E-12 | 2.12 | 1.01E-06 | 1.03 | 7.62E-01 | -1.04 | 6.92E-01 | 1.07 | 5.18E-01 |
| **Sp140** | 3.60 | 1.94E-12 | 1.75 | 3.66E-05 | 1.04 | 6.97E-01 | 1.10 | 3.70E-01 | 1.26 | 3.09E-02 |
| **Ccnd1** | 4.84 | 2.11E-12 | 1.95 | 6.14E-05 | -1.17 | 2.11E-01 | -1.12 | 3.84E-01 | -1.06 | 6.24E-01 |
| **Aldh1b1** | 3.01 | 2.15E-12 | 1.52 | 2.01E-04 | 1.05 | 5.63E-01 | -1.02 | 7.82E-01 | -1.06 | 5.07E-01 |
| **Gbp10** | 2.16 | 2.43E-12 | 1.17 | 3.34E-02 | 1.02 | 7.25E-01 | -1.03 | 6.80E-01 | 1.01 | 9.22E-01 |
| **Azi2** | 1.99 | 3.07E-12 | 1.26 | 8.67E-04 | 1.04 | 4.41E-01 | -1.00 | 9.31E-01 | 1.03 | 5.71E-01 |
| **Ppp1r15a** | 2.78 | 1.41E-10 | 1.39 | 5.60E-03 | 1.40 | 2.05E-03 | 1.38 | 2.88E-03 | 3.38 | 3.62E-12 |
| **Cd86** | 3.16 | 4.32E-12 | 1.51 | 5.72E-04 | 1.12 | 2.26E-01 | 1.02 | 8.04E-01 | 1.37 | 2.26E-03 |
| **Apol9b** | 4.71 | 4.48E-12 | 1.02 | 8.90E-01 | 1.01 | 9.51E-01 | -1.09 | 5.04E-01 | -1.08 | 5.38E-01 |
| **Ccl5** | 7.95 | 5.71E-12 | 3.56 | 5.83E-07 | 1.38 | 6.95E-02 | -1.05 | 7.79E-01 | 3.19 | 4.13E-07 |
| **Hdc** | 4.08 | 6.90E-12 | 1.40 | 1.56E-02 | 1.04 | 7.66E-01 | -1.04 | 7.21E-01 | 1.13 | 2.99E-01 |
| **Lgals9** | 4.19 | 8.03E-12 | 1.80 | 1.81E-04 | 1.13 | 3.32E-01 | -1.01 | 9.63E-01 | -1.05 | 6.72E-01 |
| **BC013712** | 3.44 | 8.36E-12 | 1.74 | 6.12E-05 | -1.10 | 3.82E-01 | 1.18 | 1.27E-01 | 1.06 | 5.49E-01 |
| **Casp4** | 2.81 | 9.83E-12 | 1.80 | 2.53E-06 | 1.11 | 2.42E-01 | 1.08 | 3.99E-01 | 1.12 | 2.09E-01 |
| **Aftph** | 2.69 | 9.86E-12 | 1.38 | 2.03E-03 | 1.08 | 3.49E-01 | 1.05 | 5.95E-01 | -1.05 | 5.79E-01 |
| **Naa25** | 2.61 | 1.03E-11 | 1.68 | 5.52E-06 | -1.01 | 8.97E-01 | -1.07 | 3.93E-01 | 1.06 | 4.85E-01 |
| **Rnf34** | 2.50 | 1.14E-11 | 1.90 | 1.06E-07 | 1.00 | 9.90E-01 | -1.05 | 5.00E-01 | 1.02 | 7.72E-01 |
| **Hsh2d** | 1.91 | 1.14E-11 | 1.29 | 3.75E-04 | 1.02 | 7.85E-01 | -1.16 | 1.13E-02 | 1.03 | 5.65E-01 |
| **Dusp28** | 2.23 | 1.74E-11 | 1.02 | 7.60E-01 | -1.00 | 9.63E-01 | 1.04 | 5.60E-01 | -1.01 | 8.80E-01 |
| **Agrn** | 3.15 | 1.81E-11 | 1.44 | 2.99E-03 | -1.07 | 4.89E-01 | 1.00 | 9.93E-01 | 1.07 | 5.06E-01 |
| **Mov10** | 3.07 | 2.07E-11 | 1.24 | 6.44E-02 | 1.00 | 9.74E-01 | -1.01 | 9.40E-01 | -1.09 | 3.82E-01 |
| **Fbxw17** | 2.81 | 2.09E-11 | 1.62 | 6.61E-05 | -1.03 | 7.60E-01 | -1.07 | 4.39E-01 | 1.04 | 6.69E-01 |
| **Rtp4** | 2.10 | 2.57E-11 | 1.38 | 1.88E-04 | -1.07 | 3.21E-01 | 1.04 | 5.28E-01 | 1.03 | 6.91E-01 |
| **Siglec1** | 3.54 | 3.23E-11 | 1.00 | 9.74E-01 | 1.05 | 6.88E-01 | 1.02 | 8.59E-01 | 1.08 | 5.18E-01 |
| **Tor1aip1** | 2.53 | 3.42E-11 | 1.61 | 2.57E-05 | -1.02 | 7.80E-01 | 1.01 | 9.48E-01 | -1.07 | 4.11E-01 |
| **Npc2** | 2.16 | 3.58E-11 | 1.10 | 2.35E-01 | 1.03 | 6.87E-01 | 1.18 | 2.47E-02 | 1.04 | 5.83E-01 |
| **Apobec1** | 3.25 | 4.27E-11 | 1.76 | 7.55E-05 | -1.03 | 8.11E-01 | 1.02 | 8.51E-01 | 1.04 | 7.29E-01 |
| **Ifi205** | 2.42 | 5.88E-11 | 1.37 | 2.15E-03 | 1.04 | 6.70E-01 | 1.04 | 6.69E-01 | 1.01 | 8.58E-01 |
| **Papd7** | 2.19 | 5.99E-11 | 1.73 | 3.85E-07 | -1.12 | 1.29E-01 | 1.06 | 3.95E-01 | -1.07 | 3.89E-01 |
| **A230050P20Rik** | 3.07 | 6.74E-11 | 1.26 | 6.11E-02 | -1.02 | 8.79E-01 | 1.01 | 9.46E-01 | -1.04 | 7.26E-01 |
| **H2-T23** | 2.64 | 6.80E-11 | 1.21 | 6.80E-02 | 1.20 | 5.13E-02 | -1.12 | 2.26E-01 | -1.08 | 4.07E-01 |
| **Ccl7** | 5.67 | 6.97E-11 | 1.21 | 2.90E-01 | 1.36 | 6.71E-02 | 1.04 | 7.94E-01 | 1.43 | 3.52E-02 |
| **Prr5l** | 1.98 | 7.88E-11 | 1.29 | 1.57E-03 | -1.05 | 4.22E-01 | -1.04 | 5.77E-01 | 1.02 | 7.26E-01 |
| **Ppp1r14d** | 1.85 | 8.29E-11 | 1.07 | 2.71E-01 | -1.05 | 4.44E-01 | -1.02 | 6.71E-01 | -1.03 | 6.00E-01 |
| **Scarf1** | 2.97 | 8.50E-11 | 1.27 | 4.58E-02 | -1.04 | 6.91E-01 | -1.10 | 3.49E-01 | -1.17 | 1.35E-01 |
| **Ppm1k** | 1.93 | 8.95E-11 | 1.26 | 2.29E-03 | 1.12 | 7.79E-02 | 1.04 | 5.02E-01 | -1.03 | 6.14E-01 |
| **Tapbpl** | 2.76 | 9.77E-11 | 1.60 | 1.81E-04 | -1.02 | 8.49E-01 | -1.05 | 6.36E-01 | -1.04 | 7.02E-01 |
| **Asb13** | 3.21 | 1.15E-10 | 1.53 | 2.10E-03 | -1.18 | 1.58E-01 | -1.15 | 2.15E-01 | -1.07 | 5.65E-01 |
| **Pou3f1** | 1.88 | 1.96E-10 | 1.07 | 3.26E-01 | -1.02 | 7.99E-01 | -1.02 | 7.02E-01 | -1.07 | 2.65E-01 |
| **1500012F01Rik** | 2.45 | 2.26E-10 | 1.49 | 3.91E-04 | 1.01 | 9.08E-01 | 1.14 | 1.36E-01 | 1.05 | 6.14E-01 |
| **Taar3** | 2.05 | 2.59E-10 | 1.53 | 1.63E-05 | 1.20 | 1.49E-02 | 1.00 | 9.87E-01 | 1.14 | 7.18E-02 |
| **Oas1a** | 2.42 | 2.63E-10 | 1.52 | 2.32E-04 | 1.08 | 4.05E-01 | -1.04 | 6.50E-01 | -1.01 | 8.95E-01 |
| **Ifi35** | 3.49 | 2.71E-10 | 1.99 | 4.08E-05 | 1.06 | 6.28E-01 | -1.10 | 4.43E-01 | 1.03 | 8.03E-01 |
| **Pml** | 3.73 | 2.93E-10 | 1.52 | 8.56E-03 | -1.14 | 3.42E-01 | 1.01 | 9.67E-01 | -1.12 | 3.86E-01 |
| **Ifitm3** | 3.78 | 3.38E-10 | 3.37 | 1.63E-08 | 1.05 | 7.27E-01 | 1.03 | 8.10E-01 | 1.05 | 7.15E-01 |
| **Tlk2** | 1.94 | 3.39E-10 | 1.09 | 2.63E-01 | 1.11 | 1.19E-01 | -1.01 | 9.10E-01 | 1.11 | 1.13E-01 |
| **5-Mar** | 2.48 | 3.52E-10 | 1.32 | 1.15E-02 | 1.04 | 7.02E-01 | -1.04 | 6.66E-01 | 1.10 | 3.23E-01 |
| **Rbm43** | 1.98 | 3.53E-10 | 1.47 | 3.44E-05 | 1.13 | 9.40E-02 | 1.27 | 1.73E-03 | -1.11 | 1.42E-01 |
| **Stxbp1** | 2.15 | 3.61E-10 | 1.11 | 2.53E-01 | -1.01 | 8.58E-01 | 1.12 | 1.39E-01 | 1.01 | 8.84E-01 |
| **Slc7a8** | 3.41 | 5.01E-10 | 1.53 | 5.82E-03 | -1.08 | 5.37E-01 | -1.04 | 7.33E-01 | -1.11 | 4.18E-01 |
| **Adap2** | 2.76 | 5.07E-10 | 1.30 | 3.13E-02 | 1.00 | 9.73E-01 | 1.07 | 5.35E-01 | 1.05 | 6.72E-01 |
| **Trafd1** | 2.96 | 6.60E-10 | 1.42 | 1.05E-02 | -1.11 | 3.74E-01 | 1.02 | 8.89E-01 | -1.03 | 8.25E-01 |
| **Gm5431** | 2.48 | 6.76E-10 | 1.85 | 4.16E-06 | -1.00 | 9.62E-01 | 1.09 | 3.75E-01 | 1.00 | 9.92E-01 |
| **Spsb1** | 1.99 | 8.19E-10 | 1.22 | 1.97E-02 | 1.02 | 7.58E-01 | 1.07 | 3.56E-01 | -1.00 | 9.71E-01 |
| **Car13** | 2.47 | 8.86E-10 | 1.33 | 1.25E-02 | -1.01 | 8.99E-01 | 1.09 | 3.88E-01 | 1.04 | 6.83E-01 |
| **Ch25h** | 3.99 | 9.11E-10 | 2.17 | 7.23E-05 | -1.01 | 9.26E-01 | -1.13 | 4.16E-01 | 1.24 | 1.56E-01 |
| **Rgs1** | 2.70 | 9.51E-10 | 1.81 | 3.17E-05 | 1.62 | 9.86E-05 | 1.21 | 8.64E-02 | 1.96 | 1.05E-06 |
| **Tmem106a** | 2.40 | 9.68E-10 | 1.17 | 1.38E-01 | -1.10 | 3.22E-01 | -1.03 | 7.81E-01 | -1.09 | 3.43E-01 |
| **Ddit3** | 3.16 | 1.02E-09 | 1.83 | 1.54E-04 | 1.66 | 3.22E-04 | 1.28 | 5.62E-02 | 2.07 | 3.04E-06 |
| **Psmb9** | 3.19 | 1.22E-09 | 1.72 | 6.30E-04 | 1.22 | 1.23E-01 | -1.09 | 5.12E-01 | 1.02 | 8.48E-01 |
| **Ccnd2** | 2.35 | 1.26E-09 | 1.70 | 2.28E-05 | 1.04 | 6.81E-01 | 1.05 | 6.06E-01 | 1.09 | 3.50E-01 |
| **Setd4** | 2.15 | 1.30E-09 | -1.15 | 1.32E-01 | 1.04 | 6.40E-01 | 1.02 | 7.89E-01 | -1.03 | 7.04E-01 |
| **Ccdc86** | 2.06 | 2.16E-09 | 1.71 | 2.62E-06 | -1.05 | 5.65E-01 | -1.02 | 8.08E-01 | -1.11 | 2.10E-01 |
| **Prpf38a** | 1.97 | 2.32E-09 | 1.15 | 1.17E-01 | 1.16 | 5.66E-02 | 1.12 | 1.47E-01 | 1.02 | 8.06E-01 |
| **Tmem184b** | 2.18 | 2.44E-09 | 1.58 | 6.87E-05 | -1.15 | 1.14E-01 | -1.09 | 3.34E-01 | -1.02 | 8.15E-01 |
| **Itpr1** | 2.68 | 2.59E-09 | 1.47 | 4.17E-03 | -1.08 | 5.08E-01 | 1.10 | 3.92E-01 | 1.06 | 5.85E-01 |
| **Ftsjd2** | 2.04 | 2.76E-09 | 1.67 | 5.25E-06 | -1.09 | 3.09E-01 | -1.13 | 1.32E-01 | 1.05 | 5.46E-01 |
| **Kynu** | 2.24 | 3.17E-09 | 1.11 | 3.31E-01 | 1.02 | 8.69E-01 | 1.03 | 7.43E-01 | 1.14 | 1.55E-01 |
| **Il6** | 1.42 | 5.36E-07 | 1.46 | 1.10E-06 | 1.35 | 6.34E-06 | 1.05 | 3.99E-01 | 1.60 | 3.54E-09 |
| **Tnfsf15** | 1.94 | 3.91E-09 | 1.07 | 4.40E-01 | -1.02 | 8.44E-01 | -1.05 | 5.22E-01 | 1.24 | 7.71E-03 |
| **Mlkl** | 3.32 | 3.92E-09 | 1.32 | 8.03E-02 | 1.03 | 8.18E-01 | -1.14 | 3.33E-01 | -1.09 | 5.50E-01 |
| **Rnf135** | 2.26 | 3.98E-09 | 1.18 | 1.20E-01 | 1.01 | 8.86E-01 | 1.08 | 4.01E-01 | 1.01 | 8.80E-01 |
| **Pcgf5** | 2.74 | 4.36E-09 | 2.09 | 5.92E-06 | -1.03 | 7.84E-01 | 1.08 | 4.92E-01 | 1.13 | 2.97E-01 |
| **Acsl1** | 3.02 | 4.81E-09 | 1.49 | 9.07E-03 | -1.12 | 3.91E-01 | -1.19 | 1.90E-01 | 1.37 | 1.96E-02 |
| **Rnf114** | 2.53 | 4.96E-09 | 1.57 | 8.44E-04 | -1.06 | 5.97E-01 | 1.04 | 7.01E-01 | 1.18 | 1.39E-01 |
| **Psmb10** | 2.45 | 5.95E-09 | 1.19 | 1.46E-01 | 1.10 | 3.89E-01 | -1.14 | 2.29E-01 | 1.06 | 6.05E-01 |
| **Asah2** | 2.26 | 6.05E-09 | 1.16 | 1.78E-01 | 1.00 | 9.73E-01 | 1.02 | 8.63E-01 | 1.02 | 8.30E-01 |
| **Ccdc23** | 2.11 | 6.29E-09 | 1.30 | 1.13E-02 | 1.06 | 5.08E-01 | -1.01 | 8.93E-01 | -1.15 | 1.24E-01 |
| **Trib3** | 2.00 | 1.31E-06 | 1.66 | 3.69E-04 | 2.56 | 6.58E-09 | 1.51 | 9.36E-04 | 2.40 | 2.56E-08 |
| **Plekha4** | 1.59 | 6.85E-09 | -1.04 | 4.93E-01 | 1.03 | 6.26E-01 | -1.01 | 8.07E-01 | 1.12 | 4.80E-02 |
| **Sgcb** | 1.87 | 7.02E-09 | 1.14 | 1.33E-01 | 1.10 | 1.90E-01 | -1.00 | 9.84E-01 | 1.00 | 9.76E-01 |
| **Setdb2** | 1.88 | 9.07E-09 | 1.29 | 5.41E-03 | 1.14 | 8.58E-02 | 1.06 | 4.36E-01 | 1.03 | 6.95E-01 |
| **Tmco3** | 1.94 | 9.16E-09 | 1.30 | 6.04E-03 | -1.20 | 3.21E-02 | -1.14 | 1.16E-01 | -1.08 | 3.53E-01 |
| **Rab7l1** | 1.83 | 9.19E-09 | 1.50 | 3.74E-05 | 1.04 | 6.18E-01 | -1.00 | 9.96E-01 | -1.03 | 6.88E-01 |
| **Csprs** | 2.31 | 9.41E-09 | -1.11 | 3.39E-01 | 1.05 | 6.12E-01 | -1.05 | 6.51E-01 | 1.13 | 2.40E-01 |
| **Hpse** | 2.05 | 9.53E-09 | 1.37 | 2.97E-03 | -1.06 | 5.14E-01 | -1.02 | 8.19E-01 | 1.05 | 5.99E-01 |
| **Il18** | 2.47 | 9.67E-09 | 1.06 | 6.26E-01 | -1.08 | 4.94E-01 | -1.01 | 9.40E-01 | 1.10 | 3.87E-01 |
| **Plekhf2** | 1.62 | 1.06E-08 | 1.09 | 1.75E-01 | -1.09 | 1.55E-01 | -1.01 | 8.55E-01 | -1.08 | 2.09E-01 |
| **Cutc** | 1.68 | 1.11E-08 | 1.15 | 5.15E-02 | 1.07 | 2.74E-01 | -1.00 | 9.90E-01 | 1.13 | 5.66E-02 |
| **Samhd1** | 2.58 | 1.28E-08 | 1.48 | 5.07E-03 | 1.13 | 3.02E-01 | -1.02 | 8.86E-01 | -1.01 | 9.15E-01 |
| **Fam82a2** | 1.88 | 1.42E-08 | 1.15 | 1.09E-01 | -1.02 | 8.21E-01 | 1.05 | 5.00E-01 | -1.02 | 7.73E-01 |
| **Tnfsf10** | 1.56 | 1.64E-08 | -1.02 | 7.55E-01 | -1.10 | 8.92E-02 | 1.03 | 5.82E-01 | -1.00 | 9.68E-01 |
| **Mfsd7a** | 1.96 | 1.93E-08 | 1.23 | 3.21E-02 | -1.00 | 9.73E-01 | -1.06 | 5.20E-01 | 1.20 | 4.01E-02 |
| **2010106G01Rik** | 1.90 | 2.27E-08 | 1.41 | 8.36E-04 | -1.01 | 8.90E-01 | 1.13 | 1.29E-01 | 1.20 | 3.40E-02 |
| **Ift172** | 1.99 | 2.30E-08 | 1.04 | 7.17E-01 | -1.01 | 9.19E-01 | -1.01 | 9.52E-01 | 1.02 | 8.52E-01 |
| **Hmgn3** | 2.44 | 2.55E-08 | 1.15 | 2.69E-01 | 1.07 | 5.55E-01 | 1.07 | 5.40E-01 | 1.01 | 9.58E-01 |
| **Slamf7** | 2.26 | 2.63E-08 | 1.33 | 2.06E-02 | 1.08 | 4.40E-01 | 1.10 | 3.67E-01 | -1.00 | 9.63E-01 |
| **Gadd45b** | 2.42 | 2.85E-08 | 1.63 | 6.25E-04 | 1.35 | 1.29E-02 | 1.10 | 4.08E-01 | 1.04 | 7.06E-01 |
| **Hbegf** | 2.00 | 2.85E-08 | 1.07 | 4.69E-01 | 1.01 | 9.26E-01 | -1.04 | 6.26E-01 | -1.03 | 7.79E-01 |
| **H2-T10** | 1.93 | 3.02E-08 | 1.31 | 7.92E-03 | -1.02 | 8.09E-01 | 1.14 | 1.21E-01 | 1.01 | 8.67E-01 |
| **Xdh** | 2.36 | 3.03E-08 | 1.19 | 1.75E-01 | -1.08 | 4.96E-01 | -1.05 | 6.78E-01 | -1.10 | 4.00E-01 |
| **Irf8** | 1.87 | 3.05E-08 | 1.26 | 1.46E-02 | 1.14 | 1.13E-01 | 1.03 | 7.04E-01 | 1.02 | 8.08E-01 |
| **Ifnab** | 1.84 | 3.25E-08 | -1.03 | 7.22E-01 | -1.01 | 8.86E-01 | -1.06 | 4.71E-01 | 1.04 | 6.21E-01 |
| **Ly6e** | 2.19 | 3.31E-08 | 1.44 | 2.92E-03 | -1.02 | 8.22E-01 | 1.13 | 2.22E-01 | 1.03 | 7.85E-01 |
| **Cd300lf** | 1.92 | 3.35E-08 | 1.28 | 1.35E-02 | -1.17 | 7.52E-02 | -1.08 | 3.63E-01 | 1.08 | 3.48E-01 |
| **Cd274** | 2.46 | 4.06E-08 | 1.28 | 6.75E-02 | 1.01 | 9.03E-01 | 1.03 | 8.23E-01 | 1.09 | 4.62E-01 |
| **Xkr8** | 1.70 | 4.21E-08 | 1.04 | 6.03E-01 | -1.02 | 7.83E-01 | -1.18 | 2.34E-02 | -1.00 | 9.64E-01 |
| **Cfb** | 4.23 | 4.27E-08 | 1.39 | 1.30E-01 | -1.05 | 7.88E-01 | -1.08 | 6.72E-01 | -1.02 | 9.29E-01 |
| **Tnfsf8** | 2.38 | 4.29E-08 | 1.45 | 6.60E-03 | -1.01 | 9.31E-01 | 1.04 | 7.01E-01 | 1.12 | 3.34E-01 |
| **Ifna4** | 1.84 | 4.55E-08 | 1.04 | 6.43E-01 | -1.02 | 8.37E-01 | -1.01 | 8.53E-01 | 1.27 | 5.60E-03 |
| **Trim56** | 1.72 | 5.40E-08 | 1.20 | 3.06E-02 | 1.09 | 2.21E-01 | -1.01 | 9.32E-01 | 1.02 | 7.55E-01 |
| **Marcksl1** | 2.00 | 6.03E-08 | 1.21 | 7.67E-02 | -1.00 | 9.68E-01 | 1.01 | 8.99E-01 | 1.33 | 4.38E-03 |
| **Ehd4** | 1.60 | 6.22E-08 | 1.03 | 6.36E-01 | -1.15 | 3.87E-02 | 1.04 | 5.47E-01 | -1.05 | 4.80E-01 |
| **Tbc1d13** | 1.83 | 6.63E-08 | 1.32 | 4.91E-03 | -1.13 | 1.36E-01 | -1.08 | 3.58E-01 | 1.00 | 9.74E-01 |
| **Nat2** | 1.72 | 6.94E-08 | 1.17 | 5.87E-02 | -1.07 | 3.46E-01 | 1.14 | 8.50E-02 | -1.05 | 4.76E-01 |
| **Nfkbiz** | 1.78 | 5.62E-07 | 2.06 | 7.55E-08 | 1.55 | 2.96E-05 | 1.23 | 2.66E-02 | 1.63 | 6.63E-06 |
| **D17Wsu92e** | 1.85 | 8.45E-08 | 1.13 | 2.11E-01 | -1.11 | 2.34E-01 | -1.12 | 1.77E-01 | -1.04 | 6.69E-01 |
| **Stk39** | 1.77 | 8.91E-08 | 1.28 | 7.63E-03 | 1.17 | 5.37E-02 | 1.19 | 3.65E-02 | -1.01 | 9.19E-01 |
| **1190002H23Rik** | 2.58 | 1.00E-07 | 1.18 | 2.67E-01 | 1.01 | 9.24E-01 | 1.09 | 5.01E-01 | 1.24 | 1.06E-01 |
| **Urgcp** | 1.72 | 1.01E-07 | 1.02 | 7.73E-01 | 1.01 | 8.88E-01 | 1.09 | 2.66E-01 | -1.09 | 2.61E-01 |
| **Clec2d** | 2.32 | 1.04E-07 | 1.72 | 2.61E-04 | 1.25 | 5.98E-02 | -1.09 | 4.64E-01 | 1.06 | 6.03E-01 |
| **H2-T24** | 2.13 | 1.17E-07 | 1.39 | 9.19E-03 | 1.08 | 4.40E-01 | 1.06 | 5.65E-01 | -1.03 | 7.84E-01 |
| **Aida** | 1.92 | 1.24E-07 | 1.39 | 2.92E-03 | -1.09 | 3.45E-01 | 1.04 | 6.62E-01 | -1.07 | 4.35E-01 |
| **Ifi203** | 1.92 | 1.32E-07 | 1.64 | 4.68E-05 | 1.07 | 4.53E-01 | 1.13 | 1.73E-01 | -1.01 | 9.05E-01 |
| **Cds1** | 1.85 | 1.32E-07 | 1.22 | 4.39E-02 | -1.05 | 5.33E-01 | 1.09 | 3.22E-01 | -1.09 | 3.30E-01 |
| **Cited4** | 2.13 | 1.39E-07 | -1.01 | 9.18E-01 | -1.09 | 3.97E-01 | 1.07 | 5.19E-01 | -1.09 | 4.17E-01 |
| **Edn1** | 3.27 | 1.45E-07 | 1.13 | 5.04E-01 | 1.11 | 5.20E-01 | 1.01 | 9.68E-01 | -1.07 | 6.72E-01 |
| **Dck** | 2.17 | 1.46E-07 | 1.47 | 3.44E-03 | 1.02 | 8.24E-01 | -1.04 | 6.87E-01 | -1.09 | 4.52E-01 |
| **Psme1** | 2.02 | 1.48E-07 | 1.34 | 1.36E-02 | 1.06 | 5.49E-01 | 1.12 | 2.69E-01 | 1.10 | 3.25E-01 |
| **Hmox2** | 1.75 | 1.50E-07 | 1.29 | 7.76E-03 | 1.04 | 6.40E-01 | 1.18 | 4.61E-02 | -1.01 | 8.95E-01 |
| **9930111J21Rik2** | 1.81 | 1.64E-07 | 1.50 | 1.85E-04 | 1.06 | 4.53E-01 | -1.07 | 4.04E-01 | -1.02 | 8.15E-01 |
| **H2-Q8** | 2.36 | 1.71E-07 | 1.13 | 3.56E-01 | 1.11 | 4.04E-01 | -1.05 | 6.78E-01 | 1.05 | 6.80E-01 |
| **Mmp13** | 3.40 | 1.87E-07 | 1.13 | 5.45E-01 | -1.01 | 9.44E-01 | 1.12 | 5.15E-01 | 1.01 | 9.47E-01 |
| **Gch1** | 2.08 | 1.88E-07 | 1.24 | 7.28E-02 | -1.02 | 8.19E-01 | -1.13 | 2.40E-01 | 1.00 | 9.64E-01 |
| **Lrp11** | 1.74 | 1.88E-07 | 1.12 | 1.88E-01 | -1.06 | 4.25E-01 | -1.12 | 1.58E-01 | 1.03 | 7.28E-01 |
| **Slfn2** | 2.54 | 2.21E-07 | 1.79 | 5.64E-04 | 1.40 | 1.81E-02 | 1.15 | 2.92E-01 | 1.08 | 5.68E-01 |
| **Fgl2** | 2.63 | 2.23E-07 | 1.23 | 1.83E-01 | 1.35 | 3.83E-02 | -1.09 | 5.34E-01 | 1.13 | 3.88E-01 |
| **Apol7c** | 2.66 | 2.24E-07 | 1.49 | 1.66E-02 | 1.30 | 7.26E-02 | 1.13 | 3.98E-01 | 1.27 | 1.01E-01 |
| **Fbrsl1** | 2.31 | 2.34E-07 | -1.01 | 9.35E-01 | -1.11 | 3.95E-01 | -1.09 | 4.86E-01 | -1.02 | 8.84E-01 |
| **Cd47** | 2.11 | 2.36E-07 | 1.77 | 6.66E-05 | -1.04 | 7.45E-01 | 1.13 | 2.58E-01 | -1.01 | 9.53E-01 |
| **Sertad3** | 1.87 | 2.45E-07 | 1.04 | 6.93E-01 | 1.09 | 3.55E-01 | -1.04 | 6.53E-01 | -1.12 | 2.34E-01 |
| **Cd180** | 2.19 | 2.62E-07 | 1.14 | 2.95E-01 | -1.16 | 2.09E-01 | -1.07 | 5.43E-01 | -1.05 | 6.75E-01 |
| **Gbp6** | 2.45 | 2.62E-07 | 1.44 | 1.71E-02 | -1.02 | 8.63E-01 | -1.14 | 3.26E-01 | 1.07 | 5.96E-01 |
| **Eng** | 2.03 | 2.84E-07 | -1.04 | 7.50E-01 | -1.08 | 4.51E-01 | -1.02 | 8.68E-01 | -1.11 | 3.15E-01 |
| **Sell** | 2.09 | 2.84E-07 | 1.30 | 3.69E-02 | 1.10 | 3.75E-01 | 1.16 | 1.74E-01 | -1.05 | 6.47E-01 |
| **Col4a2** | 1.91 | 2.99E-07 | -1.10 | 3.52E-01 | -1.10 | 3.19E-01 | -1.13 | 2.01E-01 | -1.11 | 2.89E-01 |
| **Golga3** | 1.66 | 3.36E-07 | -1.12 | 1.72E-01 | 1.01 | 8.52E-01 | -1.10 | 2.10E-01 | 1.03 | 6.46E-01 |
| **1110018G07Rik** | 2.01 | 3.43E-07 | 1.35 | 1.39E-02 | -1.08 | 4.50E-01 | -1.09 | 4.27E-01 | -1.01 | 9.30E-01 |
| **Sepw1** | 2.15 | 3.45E-07 | 1.50 | 3.36E-03 | 1.04 | 7.12E-01 | 1.03 | 8.26E-01 | -1.02 | 8.58E-01 |
| **Tnf** | 1.68 | 5.11E-07 | 1.74 | 1.08E-06 | 1.05 | 5.49E-01 | 1.05 | 5.13E-01 | 1.70 | 3.63E-07 |
| **Gbp2** | 4.49 | 3.89E-07 | 1.73 | 3.52E-02 | 1.08 | 7.42E-01 | -1.01 | 9.72E-01 | 1.39 | 1.45E-01 |
| **Sectm1a** | 1.61 | 4.36E-07 | -1.08 | 3.51E-01 | -1.12 | 1.30E-01 | 1.09 | 2.34E-01 | 1.05 | 5.27E-01 |
| **Slfn10-ps** | 1.50 | 4.37E-07 | 1.02 | 7.23E-01 | -1.04 | 5.61E-01 | -1.03 | 6.54E-01 | 1.12 | 6.38E-02 |
| **H2-D1** | 2.02 | 4.39E-07 | 1.09 | 4.71E-01 | 1.13 | 2.46E-01 | 1.07 | 4.95E-01 | 1.06 | 5.75E-01 |
| **Tap2** | 1.83 | 4.90E-07 | -1.03 | 7.52E-01 | 1.10 | 3.12E-01 | 1.05 | 5.77E-01 | -1.02 | 8.68E-01 |
| **Casp1** | 1.91 | 4.97E-07 | 1.25 | 4.81E-02 | -1.12 | 2.64E-01 | -1.09 | 3.66E-01 | 1.02 | 8.32E-01 |
| **C130026I21Rik** | 1.91 | 5.81E-07 | 1.11 | 3.55E-01 | 1.12 | 2.48E-01 | 1.01 | 9.00E-01 | 1.05 | 6.16E-01 |
| **Sdc3** | 2.04 | 6.10E-07 | 1.64 | 3.52E-04 | -1.05 | 6.73E-01 | -1.09 | 4.31E-01 | -1.07 | 5.39E-01 |
| **Chmp4b** | 1.80 | 6.20E-07 | 1.03 | 7.76E-01 | 1.03 | 7.04E-01 | -1.09 | 3.38E-01 | 1.05 | 5.56E-01 |
| **Bst2** | 4.16 | 6.35E-07 | -1.13 | 6.06E-01 | 1.16 | 4.99E-01 | -1.54 | 5.59E-02 | -1.01 | 9.53E-01 |
| **Dennd3** | 1.84 | 6.62E-07 | 1.02 | 8.44E-01 | -1.06 | 5.06E-01 | 1.11 | 2.82E-01 | 1.00 | 9.85E-01 |
| **Ankle2** | 1.63 | 6.88E-07 | 1.18 | 6.13E-02 | -1.09 | 2.33E-01 | 1.05 | 5.10E-01 | -1.05 | 4.88E-01 |
| **Ifna9** | 1.59 | 6.98E-07 | 1.01 | 8.82E-01 | 1.03 | 6.91E-01 | -1.09 | 2.24E-01 | 1.15 | 5.70E-02 |
| **Ap3m2** | 1.67 | 7.01E-07 | 1.00 | 9.67E-01 | -1.06 | 4.44E-01 | -1.13 | 1.20E-01 | -1.05 | 5.32E-01 |
| **Usp25** | 1.57 | 7.03E-07 | 1.18 | 3.78E-02 | 1.04 | 5.58E-01 | -1.14 | 6.69E-02 | 1.08 | 2.58E-01 |
| **Sh3bp2** | 1.53 | 7.76E-07 | -1.12 | 1.42E-01 | -1.07 | 3.10E-01 | -1.00 | 9.70E-01 | 1.01 | 9.23E-01 |
| **Akt3** | 1.97 | 7.81E-07 | 1.04 | 7.23E-01 | 1.16 | 1.61E-01 | -1.11 | 3.15E-01 | 1.03 | 7.69E-01 |
| **Ogfrl1** | 1.81 | 8.35E-07 | 1.36 | 5.97E-03 | -1.00 | 9.90E-01 | -1.13 | 2.03E-01 | 1.05 | 6.14E-01 |
| **Rell1** | 1.50 | 8.95E-07 | -1.01 | 9.40E-01 | 1.07 | 2.86E-01 | 1.10 | 1.47E-01 | 1.06 | 3.72E-01 |
| **Brdt** | 1.51 | 1.08E-06 | 1.07 | 3.37E-01 | 1.06 | 3.98E-01 | -1.01 | 8.61E-01 | -1.06 | 3.52E-01 |
| **Sertad1** | 1.51 | 1.11E-06 | 1.16 | 4.93E-02 | 1.02 | 7.68E-01 | 1.08 | 2.33E-01 | -1.12 | 9.91E-02 |
| **Keap1** | 1.99 | 1.18E-06 | 1.19 | 1.61E-01 | 1.08 | 5.03E-01 | -1.13 | 2.53E-01 | -1.13 | 2.55E-01 |
| **Fam53c** | 1.74 | 1.27E-06 | 1.08 | 4.49E-01 | -1.02 | 8.44E-01 | -1.01 | 9.46E-01 | -1.04 | 6.31E-01 |
| **Csrnp1** | 1.59 | 1.34E-06 | 1.11 | 2.04E-01 | 1.02 | 8.06E-01 | 1.03 | 6.53E-01 | 1.32 | 8.81E-04 |
| **Tlr8** | 2.03 | 1.38E-06 | 1.18 | 1.91E-01 | -1.24 | 6.91E-02 | -1.24 | 6.36E-02 | -1.10 | 4.22E-01 |
| **Ccr7** | 2.09 | 1.44E-06 | 1.40 | 1.66E-02 | 1.14 | 2.70E-01 | 1.03 | 7.85E-01 | 1.21 | 1.13E-01 |
| **Cxcl2** | 1.10 | 4.43E-01 | 1.59 | 2.98E-03 | 2.20 | 1.56E-06 | 1.32 | 3.56E-02 | 1.37 | 1.93E-02 |
| **Cox18** | 1.80 | 1.65E-06 | 1.22 | 6.86E-02 | 1.01 | 9.37E-01 | -1.08 | 4.23E-01 | 1.04 | 6.50E-01 |
| **Tcf4** | 1.67 | 1.79E-06 | 1.25 | 2.49E-02 | 1.00 | 9.71E-01 | -1.21 | 2.75E-02 | 1.02 | 8.01E-01 |
| **Gypc** | 1.71 | 1.82E-06 | 1.11 | 2.93E-01 | -1.03 | 7.50E-01 | 1.02 | 8.05E-01 | 1.23 | 2.35E-02 |
| **Chac1** | 1.48 | 6.55E-04 | 1.29 | 3.49E-02 | 1.63 | 5.87E-05 | 1.16 | 1.56E-01 | 1.87 | 1.86E-06 |
| **Dbnl** | 1.70 | 1.86E-06 | 1.17 | 1.16E-01 | 1.03 | 7.16E-01 | 1.07 | 4.59E-01 | 1.02 | 8.09E-01 |
| **Tmem229b** | 1.60 | 2.04E-06 | 1.21 | 3.38E-02 | 1.01 | 9.13E-01 | -1.03 | 7.01E-01 | -1.03 | 7.19E-01 |
| **Carhsp1** | 1.86 | 2.22E-06 | 1.08 | 5.29E-01 | -1.11 | 3.34E-01 | -1.05 | 6.47E-01 | -1.08 | 4.77E-01 |
| **Peli1** | 1.85 | 2.27E-06 | 1.39 | 7.82E-03 | 1.03 | 7.61E-01 | 1.10 | 3.61E-01 | 1.17 | 1.39E-01 |
| **Rnf31** | 1.82 | 2.30E-06 | 1.26 | 4.24E-02 | -1.01 | 8.98E-01 | 1.02 | 8.12E-01 | -1.02 | 8.47E-01 |
| **Rasgrp1** | 1.80 | 2.71E-06 | 1.16 | 1.84E-01 | -1.04 | 7.07E-01 | 1.10 | 3.45E-01 | 1.13 | 2.18E-01 |
| **Mertk** | 2.28 | 2.81E-06 | 1.21 | 2.31E-01 | -1.22 | 1.65E-01 | 1.07 | 6.06E-01 | 1.04 | 7.86E-01 |
| **Chpt1** | 2.20 | 2.81E-06 | 1.43 | 2.18E-02 | 1.01 | 9.43E-01 | -1.16 | 2.80E-01 | 1.01 | 9.22E-01 |
| **Serpina3g** | 3.37 | 2.83E-06 | 1.44 | 1.20E-01 | 1.21 | 3.47E-01 | -1.02 | 9.42E-01 | 1.14 | 5.18E-01 |
| **Atf3** | 1.86 | 2.84E-06 | 1.36 | 1.30E-02 | 1.20 | 8.50E-02 | 1.05 | 6.36E-01 | 1.15 | 1.85E-01 |
| **Pik3ap1** | 1.89 | 2.97E-06 | 1.35 | 1.93E-02 | -1.03 | 7.94E-01 | 1.01 | 9.53E-01 | -1.01 | 9.45E-01 |
| **Tor1aip2** | 1.68 | 3.12E-06 | 1.42 | 1.41E-03 | -1.10 | 2.76E-01 | -1.04 | 6.37E-01 | -1.04 | 6.28E-01 |
| **Tspo** | 1.86 | 3.19E-06 | 1.10 | 4.29E-01 | 1.05 | 6.31E-01 | -1.10 | 3.73E-01 | 1.10 | 3.83E-01 |
| **Gpr141** | 2.08 | 3.24E-06 | 1.32 | 5.38E-02 | -1.15 | 2.62E-01 | -1.01 | 9.68E-01 | 1.02 | 8.76E-01 |
| **B430306N03Rik** | 1.67 | 3.32E-06 | 1.03 | 7.84E-01 | -1.06 | 4.93E-01 | -1.03 | 7.51E-01 | 1.03 | 7.38E-01 |
| **Nupr1** | 1.96 | 3.34E-06 | 1.44 | 7.67E-03 | 1.43 | 4.12E-03 | 1.29 | 3.42E-02 | 1.91 | 5.95E-06 |
| **Gm8909** | 1.85 | 3.77E-06 | -1.02 | 8.68E-01 | 1.02 | 8.65E-01 | -1.05 | 6.50E-01 | -1.05 | 6.44E-01 |
| **Lass6** | 1.75 | 3.80E-06 | 1.04 | 6.93E-01 | -1.08 | 4.07E-01 | 1.15 | 1.61E-01 | 1.01 | 9.50E-01 |
| **Zfp36** | 1.51 | 4.03E-06 | 1.14 | 1.09E-01 | -1.03 | 6.47E-01 | 1.15 | 5.32E-02 | -1.05 | 5.20E-01 |
| **Max** | 1.58 | 4.19E-06 | 1.10 | 2.75E-01 | 1.05 | 5.69E-01 | 1.17 | 5.87E-02 | 1.05 | 5.56E-01 |
| **Aim1** | 1.96 | 4.26E-06 | 1.41 | 1.29E-02 | -1.18 | 1.66E-01 | -1.10 | 3.99E-01 | 1.02 | 8.46E-01 |
| **Triobp** | 1.63 | 4.48E-06 | -1.00 | 9.69E-01 | -1.02 | 8.37E-01 | 1.06 | 5.03E-01 | 1.01 | 8.79E-01 |
| **Igf2bp2** | 1.94 | 4.60E-06 | 1.12 | 3.72E-01 | -1.01 | 9.44E-01 | -1.09 | 4.73E-01 | -1.04 | 7.63E-01 |
| **Trim72** | 1.59 | 4.81E-06 | -1.05 | 6.15E-01 | -1.00 | 9.86E-01 | 1.10 | 2.31E-01 | -1.04 | 6.51E-01 |
| **Rab3d** | 1.77 | 4.81E-06 | 1.26 | 4.73E-02 | -1.00 | 9.69E-01 | -1.04 | 6.89E-01 | -1.05 | 6.25E-01 |
| **Lgals8** | 2.08 | 4.82E-06 | 1.37 | 3.62E-02 | -1.13 | 3.39E-01 | -1.00 | 9.77E-01 | 1.01 | 9.08E-01 |
| **Ddhd1** | 1.77 | 4.96E-06 | 1.17 | 1.59E-01 | -1.16 | 1.55E-01 | 1.07 | 4.89E-01 | 1.09 | 3.97E-01 |
| **Unc93b1** | 1.56 | 5.08E-06 | 1.20 | 4.60E-02 | -1.07 | 3.71E-01 | -1.07 | 4.14E-01 | -1.00 | 9.65E-01 |
| **Bbx** | 1.60 | 5.08E-06 | 1.02 | 8.55E-01 | -1.01 | 8.63E-01 | 1.06 | 5.06E-01 | 1.08 | 3.79E-01 |
| **Mxd1** | 1.52 | 5.13E-06 | 1.06 | 4.85E-01 | -1.04 | 5.50E-01 | 1.06 | 4.41E-01 | 1.06 | 4.22E-01 |
| **Il10ra** | 1.63 | 5.77E-06 | 1.46 | 5.88E-04 | -1.02 | 7.78E-01 | 1.01 | 8.87E-01 | -1.00 | 9.59E-01 |
| **Traf1** | 1.99 | 5.95E-06 | 1.43 | 1.43E-02 | 1.27 | 5.92E-02 | 1.13 | 3.25E-01 | 1.27 | 5.95E-02 |
| **Psmb8** | 1.86 | 6.38E-06 | 1.30 | 4.09E-02 | 1.23 | 6.72E-02 | 1.06 | 5.88E-01 | 1.01 | 9.23E-01 |
| **6330578E17Rik** | 1.63 | 6.80E-06 | 1.15 | 1.62E-01 | -1.08 | 4.04E-01 | 1.03 | 7.10E-01 | 1.03 | 7.65E-01 |
| **Sdc4** | 1.45 | 4.51E-04 | 1.01 | 9.51E-01 | -1.05 | 6.09E-01 | -1.00 | 9.92E-01 | 1.69 | 6.98E-06 |
| **G3bp2** | 1.74 | 7.72E-06 | 1.14 | 2.44E-01 | -1.14 | 2.01E-01 | 1.13 | 2.36E-01 | -1.01 | 9.23E-01 |
| **Rgs16** | 1.63 | 8.31E-06 | -1.06 | 5.76E-01 | -1.01 | 9.23E-01 | 1.06 | 4.85E-01 | 1.01 | 8.73E-01 |
| **Il27** | 1.67 | 8.80E-06 | 1.11 | 3.41E-01 | 1.06 | 5.07E-01 | -1.11 | 2.72E-01 | 1.03 | 7.32E-01 |
| **Otud5** | 1.72 | 8.84E-06 | 1.27 | 3.97E-02 | 1.14 | 2.01E-01 | 1.07 | 4.98E-01 | 1.05 | 6.22E-01 |
| **Ccl2** | 2.45 | 9.08E-06 | 1.57 | 1.96E-02 | 1.34 | 7.83E-02 | 1.11 | 5.13E-01 | 1.13 | 4.41E-01 |
| **Lpxn** | 1.55 | 9.30E-06 | 1.16 | 9.53E-02 | 1.08 | 3.29E-01 | 1.02 | 8.51E-01 | -1.11 | 2.03E-01 |
| **Ccl4** | 1.61 | 9.66E-06 | 1.26 | 2.52E-02 | 1.20 | 4.70E-02 | -1.00 | 9.98E-01 | 1.13 | 1.74E-01 |
| **Aff1** | 1.53 | 9.68E-06 | -1.00 | 9.59E-01 | 1.05 | 5.36E-01 | -1.02 | 7.78E-01 | -1.12 | 1.64E-01 |
| **Zbp1** | 1.63 | 1.17E-05 | 1.41 | 2.19E-03 | 1.05 | 5.98E-01 | -1.04 | 6.95E-01 | -1.02 | 7.95E-01 |
| **BC005537** | 1.51 | 1.32E-05 | 1.08 | 3.95E-01 | 1.00 | 9.91E-01 | 1.14 | 1.05E-01 | 1.06 | 4.19E-01 |
| **Papd4** | 1.68 | 1.66E-05 | 1.34 | 1.28E-02 | -1.01 | 9.16E-01 | 1.17 | 1.30E-01 | 1.10 | 3.54E-01 |
| **Ankfy1** | 1.61 | 1.69E-05 | 1.03 | 7.40E-01 | -1.06 | 5.04E-01 | 1.08 | 3.93E-01 | -1.10 | 3.04E-01 |
| **Morc3** | 1.50 | 1.76E-05 | 1.29 | 7.10E-03 | -1.06 | 4.47E-01 | 1.01 | 8.50E-01 | -1.02 | 7.77E-01 |
| **Atp10a** | 1.90 | 1.81E-05 | 1.55 | 3.62E-03 | 1.00 | 9.70E-01 | -1.00 | 9.91E-01 | -1.18 | 1.75E-01 |
| **Htra2** | 1.61 | 1.95E-05 | 1.38 | 4.32E-03 | -1.06 | 5.07E-01 | 1.08 | 4.19E-01 | 1.03 | 7.66E-01 |
| **Pcsk7** | 1.67 | 1.98E-05 | 1.06 | 5.84E-01 | -1.04 | 7.10E-01 | -1.08 | 4.63E-01 | -1.06 | 5.86E-01 |
| **Tgif1** | 1.57 | 2.01E-05 | 1.12 | 2.39E-01 | 1.29 | 6.73E-03 | 1.22 | 2.77E-02 | 1.23 | 2.28E-02 |
| **Zcchc6** | 1.63 | 2.10E-05 | -1.02 | 8.65E-01 | -1.09 | 3.72E-01 | -1.10 | 3.18E-01 | 1.00 | 9.71E-01 |
| **Plin2** | 1.87 | 2.16E-05 | 1.25 | 1.08E-01 | 1.20 | 1.44E-01 | 1.02 | 8.93E-01 | 1.14 | 2.76E-01 |
| **Etv3** | 1.54 | 2.20E-05 | 1.15 | 1.37E-01 | 1.16 | 8.40E-02 | 1.08 | 3.85E-01 | 1.14 | 1.39E-01 |
| **Plod3** | 1.67 | 2.40E-05 | -1.08 | 4.87E-01 | -1.07 | 4.87E-01 | 1.03 | 7.96E-01 | 1.02 | 8.70E-01 |
| **Slc7a2** | 2.14 | 2.45E-05 | 1.14 | 4.32E-01 | -1.12 | 4.63E-01 | -1.00 | 9.80E-01 | 1.03 | 8.55E-01 |
| **Rab21** | 1.60 | 2.65E-05 | 1.12 | 2.83E-01 | -1.02 | 7.92E-01 | -1.02 | 8.33E-01 | -1.06 | 5.09E-01 |
| **H2-M3** | 1.71 | 2.66E-05 | 1.34 | 1.97E-02 | -1.03 | 7.77E-01 | -1.01 | 8.91E-01 | 1.03 | 7.96E-01 |
| **Mthfd2** | 1.58 | 2.72E-05 | -1.06 | 5.51E-01 | 1.24 | 2.52E-02 | 1.03 | 7.16E-01 | 1.20 | 5.45E-02 |
| **Ripk2** | 1.62 | 2.78E-05 | 1.23 | 5.82E-02 | 1.24 | 3.10E-02 | -1.07 | 5.02E-01 | -1.00 | 9.95E-01 |
| **Clcn7** | 1.69 | 2.93E-05 | 1.25 | 6.17E-02 | -1.04 | 6.79E-01 | 1.08 | 4.82E-01 | 1.01 | 9.07E-01 |
| **Tsc22d1** | 1.53 | 2.95E-05 | 1.01 | 9.21E-01 | -1.03 | 6.96E-01 | 1.02 | 8.40E-01 | -1.03 | 7.58E-01 |
| **Ly6c1** | 2.84 | 3.01E-05 | 1.74 | 2.41E-02 | 1.08 | 7.25E-01 | -1.00 | 9.84E-01 | -1.05 | 8.16E-01 |
| **Gtpbp2** | 1.69 | 3.27E-05 | 1.18 | 1.64E-01 | 1.03 | 7.79E-01 | -1.00 | 9.64E-01 | 1.29 | 2.05E-02 |
| **Slamf8** | 2.38 | 3.58E-05 | 1.25 | 2.59E-01 | 1.21 | 2.87E-01 | 1.07 | 7.01E-01 | 1.10 | 5.83E-01 |
| **Cept1** | 1.51 | 3.61E-05 | 1.05 | 5.84E-01 | -1.05 | 5.95E-01 | 1.05 | 5.58E-01 | 1.13 | 1.41E-01 |
| **Plk2** | 1.75 | 3.68E-05 | 1.08 | 5.68E-01 | 1.29 | 3.15E-02 | 1.23 | 7.58E-02 | 1.17 | 1.84E-01 |
| **Cebpe** | 2.24 | 3.89E-05 | 1.52 | 2.97E-02 | 1.14 | 4.28E-01 | 1.07 | 6.99E-01 | 1.11 | 5.28E-01 |
| **Ppa1** | 1.55 | 4.23E-05 | 1.15 | 1.71E-01 | -1.04 | 6.82E-01 | -1.01 | 8.81E-01 | 1.04 | 6.27E-01 |
| **Bzrap1** | 2.04 | 4.37E-05 | 1.27 | 1.53E-01 | -1.10 | 5.19E-01 | 1.16 | 3.15E-01 | 1.20 | 2.18E-01 |
| **Parp3** | 1.74 | 4.48E-05 | 1.08 | 5.69E-01 | 1.07 | 5.42E-01 | 1.05 | 6.83E-01 | 1.02 | 8.82E-01 |
| **Inhba** | 2.30 | 4.52E-05 | 1.02 | 9.36E-01 | 1.07 | 6.80E-01 | 1.06 | 7.45E-01 | 1.17 | 3.54E-01 |
| **Flrt2** | 1.92 | 4.60E-05 | 1.07 | 6.61E-01 | -1.01 | 9.44E-01 | -1.11 | 4.53E-01 | 1.02 | 8.92E-01 |
| **Osm** | 1.56 | 4.65E-05 | 1.18 | 1.16E-01 | -1.02 | 8.33E-01 | 1.05 | 5.62E-01 | -1.09 | 3.59E-01 |
| **Dpp4** | 1.82 | 4.69E-05 | 1.34 | 4.28E-02 | 1.01 | 9.22E-01 | 1.02 | 8.72E-01 | -1.03 | 7.98E-01 |
| **Tapbp** | 2.82 | 4.76E-05 | 1.30 | 2.77E-01 | 1.07 | 7.57E-01 | -1.09 | 6.85E-01 | -1.05 | 8.20E-01 |
| **Cited2** | 1.97 | 4.96E-05 | 1.20 | 2.46E-01 | -1.07 | 6.36E-01 | -1.13 | 3.97E-01 | 1.13 | 3.92E-01 |
| **Grasp** | 1.52 | 5.25E-05 | 1.05 | 5.87E-01 | 1.15 | 1.16E-01 | 1.06 | 5.25E-01 | 1.18 | 6.58E-02 |
| **Ddx24** | 1.67 | 5.31E-05 | 1.36 | 1.46E-02 | 1.05 | 6.44E-01 | -1.18 | 1.36E-01 | -1.08 | 4.70E-01 |
| **Trim26** | 1.62 | 5.62E-05 | 1.25 | 5.92E-02 | -1.07 | 5.29E-01 | 1.06 | 5.54E-01 | 1.09 | 3.87E-01 |
| **Slc12a9** | 1.58 | 5.74E-05 | 1.03 | 7.59E-01 | -1.01 | 9.45E-01 | -1.15 | 1.60E-01 | 1.03 | 7.26E-01 |
| **Ncoa1** | 1.52 | 5.83E-05 | -1.15 | 1.60E-01 | 1.03 | 7.73E-01 | -1.12 | 2.18E-01 | -1.11 | 2.33E-01 |
| **Pdcd1lg2** | 1.52 | 6.60E-05 | 1.06 | 5.46E-01 | -1.04 | 6.70E-01 | 1.17 | 8.38E-02 | -1.02 | 8.65E-01 |
| **Nmral1** | 1.62 | 6.61E-05 | 1.36 | 1.17E-02 | -1.06 | 5.90E-01 | -1.25 | 3.54E-02 | -1.04 | 7.08E-01 |
| **Gnb4** | 1.57 | 6.79E-05 | 1.28 | 2.82E-02 | -1.05 | 6.01E-01 | 1.10 | 3.02E-01 | 1.10 | 3.15E-01 |
| **Rab19** | 1.65 | 6.83E-05 | 1.14 | 2.65E-01 | -1.04 | 6.97E-01 | -1.02 | 8.22E-01 | -1.09 | 4.06E-01 |
| **Whamm** | 1.51 | 6.90E-05 | 1.23 | 3.95E-02 | 1.17 | 9.05E-02 | -1.11 | 2.43E-01 | -1.01 | 9.17E-01 |
| **Stard3** | 1.65 | 7.01E-05 | 1.13 | 3.25E-01 | 1.23 | 5.63E-02 | -1.22 | 6.93E-02 | 1.00 | 9.69E-01 |
| **Arid5b** | 1.50 | 7.40E-05 | 1.10 | 3.19E-01 | 1.04 | 6.50E-01 | 1.05 | 5.97E-01 | 1.06 | 4.84E-01 |
| **Cxcl1** | -1.11 | 5.33E-01 | 1.26 | 2.25E-01 | 2.22 | 7.46E-05 | -1.07 | 7.04E-01 | 1.27 | 1.68E-01 |
| **Kif1a** | 1.52 | 7.59E-05 | -1.02 | 8.36E-01 | 1.17 | 9.65E-02 | 1.02 | 8.31E-01 | 1.02 | 8.18E-01 |
| **Ap3b1** | 1.51 | 7.85E-05 | -1.03 | 7.62E-01 | 1.01 | 9.31E-01 | -1.00 | 9.64E-01 | -1.03 | 6.97E-01 |
| **P2ry14** | 1.67 | 8.02E-05 | 1.18 | 1.80E-01 | -1.11 | 3.55E-01 | 1.09 | 4.15E-01 | 1.01 | 9.01E-01 |
| **Flii** | 1.50 | 8.55E-05 | -1.20 | 7.18E-02 | 1.04 | 6.53E-01 | -1.17 | 7.52E-02 | -1.01 | 8.81E-01 |
| **Naa20** | 1.17 | 7.11E-02 | 1.56 | 8.88E-05 | 1.03 | 7.67E-01 | -1.06 | 5.27E-01 | 1.03 | 7.67E-01 |
| **Hdac1** | 1.55 | 1.03E-04 | 1.00 | 9.87E-01 | -1.01 | 9.36E-01 | -1.05 | 5.81E-01 | -1.01 | 8.92E-01 |
| **5031414D18Rik** | 1.54 | 1.13E-04 | 1.08 | 4.70E-01 | 1.01 | 8.92E-01 | 1.10 | 3.45E-01 | -1.03 | 7.95E-01 |
| **Ptpn6** | 1.52 | 1.26E-04 | -1.05 | 6.21E-01 | 1.17 | 9.81E-02 | 1.15 | 1.37E-01 | 1.10 | 3.19E-01 |
| **Tgm2** | 1.83 | 1.31E-04 | 1.06 | 6.87E-01 | 1.06 | 6.85E-01 | -1.12 | 4.19E-01 | 1.14 | 3.38E-01 |
| **Mid1** | 1.86 | 1.47E-04 | 1.23 | 1.93E-01 | -1.07 | 6.36E-01 | 1.07 | 6.36E-01 | -1.09 | 5.53E-01 |
| **Ptpro** | 1.51 | 1.58E-04 | 1.03 | 7.67E-01 | -1.06 | 5.03E-01 | -1.02 | 8.06E-01 | 1.22 | 4.47E-02 |
| **Aif1** | 1.56 | 1.59E-04 | 1.08 | 4.88E-01 | 1.28 | 2.30E-02 | -1.18 | 1.06E-01 | 1.01 | 9.45E-01 |
| **Rnpep** | 1.50 | 1.60E-04 | -1.03 | 7.54E-01 | -1.08 | 4.18E-01 | -1.15 | 1.40E-01 | -1.06 | 5.58E-01 |
| **Il1rn** | 1.87 | 1.61E-04 | 1.01 | 9.65E-01 | 1.07 | 6.52E-01 | -1.10 | 5.20E-01 | 1.07 | 6.60E-01 |
| **Insl6** | 1.81 | 1.63E-04 | 1.09 | 5.71E-01 | 1.07 | 6.23E-01 | -1.04 | 7.97E-01 | -1.02 | 8.81E-01 |
| **Gsdmd** | 1.54 | 1.63E-04 | 1.03 | 8.21E-01 | 1.01 | 9.52E-01 | 1.07 | 4.64E-01 | 1.22 | 5.00E-02 |
| **Lrrk2** | 1.52 | 1.67E-04 | 1.12 | 3.02E-01 | -1.06 | 5.71E-01 | 1.02 | 8.64E-01 | -1.05 | 5.95E-01 |
| **Ush2a** | 1.53 | 1.67E-04 | 1.07 | 5.14E-01 | 1.25 | 2.87E-02 | 1.00 | 9.62E-01 | 1.09 | 3.96E-01 |
| **Cd226** | 1.61 | 1.70E-04 | 1.15 | 2.53E-01 | 1.06 | 5.91E-01 | 1.25 | 4.55E-02 | -1.01 | 9.32E-01 |
| **Il12rb2** | 1.63 | 1.75E-04 | 1.28 | 6.16E-02 | -1.02 | 8.62E-01 | -1.02 | 8.64E-01 | 1.14 | 2.45E-01 |
| **Csf1** | 1.71 | 1.81E-04 | 1.06 | 6.76E-01 | 1.19 | 1.61E-01 | 1.09 | 4.93E-01 | 1.05 | 6.99E-01 |
| **Tmem50a** | 1.06 | 6.52E-01 | 1.05 | 7.36E-01 | -1.10 | 4.23E-01 | 1.70 | 2.15E-04 | -1.14 | 3.00E-01 |
| **Slc29a3** | 1.56 | 2.19E-04 | -1.20 | 1.35E-01 | -1.09 | 4.23E-01 | -1.00 | 9.70E-01 | -1.10 | 3.69E-01 |
| **Wars** | 1.61 | 2.30E-04 | 1.12 | 3.64E-01 | 1.08 | 5.00E-01 | 1.16 | 1.96E-01 | 1.28 | 3.68E-02 |
| **Ikbke** | 1.61 | 2.87E-04 | 1.21 | 1.49E-01 | -1.16 | 1.98E-01 | -1.00 | 9.95E-01 | 1.05 | 6.87E-01 |
| **Tank** | 1.55 | 2.91E-04 | 1.20 | 1.32E-01 | -1.02 | 8.51E-01 | 1.10 | 3.85E-01 | 1.14 | 2.25E-01 |
| **Tlr7** | 1.82 | 2.92E-04 | 1.23 | 2.10E-01 | -1.14 | 3.73E-01 | -1.00 | 9.75E-01 | 1.00 | 9.96E-01 |
| **Sema7a** | 1.58 | 3.13E-04 | -1.01 | 9.26E-01 | 1.23 | 7.03E-02 | 1.01 | 9.31E-01 | -1.01 | 9.24E-01 |
| **Tbc1d8** | 1.52 | 3.15E-04 | 1.33 | 1.89E-02 | 1.16 | 1.42E-01 | 1.21 | 7.18E-02 | 1.19 | 9.45E-02 |
| **Tnfrsf1b** | 1.69 | 3.40E-04 | 1.03 | 8.24E-01 | 1.09 | 5.23E-01 | -1.02 | 8.80E-01 | -1.00 | 9.84E-01 |
| **Shisa5** | 1.54 | 3.61E-04 | 1.27 | 4.99E-02 | 1.09 | 3.92E-01 | 1.03 | 7.78E-01 | -1.06 | 5.57E-01 |
| **Rrbp1** | 1.56 | 3.61E-04 | 1.13 | 3.24E-01 | 1.03 | 7.68E-01 | 1.05 | 6.82E-01 | 1.04 | 7.42E-01 |
| **Birc2** | 1.54 | 3.93E-04 | 1.43 | 5.25E-03 | -1.02 | 8.28E-01 | 1.10 | 3.81E-01 | -1.00 | 9.77E-01 |
| **Atp6v0a2** | 1.53 | 3.99E-04 | 1.05 | 6.70E-01 | -1.16 | 1.55E-01 | 1.09 | 3.94E-01 | 1.05 | 6.27E-01 |
| **Apobec3** | 1.56 | 4.09E-04 | 1.51 | 2.58E-03 | -1.01 | 8.99E-01 | 1.20 | 1.13E-01 | -1.01 | 9.55E-01 |
| **Il1a** | 1.86 | 4.15E-04 | 1.74 | 3.25E-03 | 1.10 | 5.34E-01 | 1.14 | 3.84E-01 | 1.48 | 1.65E-02 |
| **Fscn1** | 2.30 | 4.18E-04 | 2.06 | 4.05E-03 | 1.08 | 7.14E-01 | 1.11 | 6.17E-01 | -1.02 | 9.12E-01 |
| **Naip5** | 1.52 | 4.27E-04 | 1.11 | 3.68E-01 | -1.20 | 8.90E-02 | -1.23 | 5.82E-02 | -1.05 | 6.35E-01 |
| **Gm885** | 1.56 | 4.46E-04 | 1.13 | 3.25E-01 | 1.18 | 1.38E-01 | 1.01 | 9.14E-01 | -1.11 | 3.66E-01 |
| **Hk3** | 1.72 | 5.13E-04 | -1.10 | 5.32E-01 | -1.08 | 5.96E-01 | -1.04 | 7.79E-01 | -1.10 | 4.73E-01 |
| **Ly86** | 2.18 | 5.42E-04 | 1.31 | 2.27E-01 | 1.18 | 4.09E-01 | 1.04 | 8.48E-01 | -1.01 | 9.76E-01 |
| **Rasgef1b** | 1.55 | 5.64E-04 | 1.31 | 4.25E-02 | -1.09 | 4.64E-01 | 1.02 | 8.69E-01 | -1.02 | 8.27E-01 |
| **Mpp1** | 1.90 | 5.76E-04 | -1.07 | 7.14E-01 | -1.11 | 5.41E-01 | -1.09 | 6.12E-01 | 1.09 | 5.84E-01 |
| **Pi4k2a** | 1.55 | 5.83E-04 | -1.16 | 2.44E-01 | -1.05 | 6.47E-01 | -1.04 | 7.59E-01 | -1.04 | 7.49E-01 |
| **Gadd45a** | -1.01 | 9.11E-01 | 1.73 | 9.07E-04 | 1.67 | 6.18E-04 | 1.24 | 1.15E-01 | 1.66 | 6.86E-04 |
| **Cdkn1a** | 1.66 | 6.35E-04 | 1.40 | 2.79E-02 | 1.27 | 7.56E-02 | 1.09 | 5.25E-01 | -1.07 | 5.92E-01 |
| **2310016C08Rik** | 1.57 | 6.77E-04 | 1.04 | 7.85E-01 | 1.03 | 7.82E-01 | 1.17 | 1.88E-01 | 1.29 | 3.91E-02 |
| **Sgk1** | 2.17 | 6.92E-04 | 1.17 | 4.93E-01 | -1.08 | 6.88E-01 | -1.09 | 6.69E-01 | 1.07 | 7.22E-01 |
| **Gm7609** | 1.63 | 7.35E-04 | -1.01 | 9.70E-01 | 1.13 | 3.48E-01 | -1.01 | 9.12E-01 | 1.03 | 8.36E-01 |
| **Tmem2** | 1.55 | 7.43E-04 | 1.05 | 6.79E-01 | -1.12 | 3.19E-01 | 1.05 | 6.87E-01 | -1.16 | 2.03E-01 |
| **Rap2c** | 1.61 | 7.71E-04 | 1.22 | 1.67E-01 | -1.03 | 8.17E-01 | -1.02 | 8.98E-01 | 1.05 | 7.21E-01 |
| **Pdk3** | 1.70 | 7.92E-04 | 1.32 | 8.59E-02 | 1.08 | 5.68E-01 | -1.03 | 8.56E-01 | -1.12 | 4.14E-01 |
| **Samsn1** | 1.02 | 8.43E-01 | 1.52 | 8.89E-04 | 1.07 | 5.23E-01 | 1.09 | 4.20E-01 | 1.25 | 3.72E-02 |
| **Cd83** | 1.59 | 2.06E-03 | 1.75 | 1.07E-03 | 1.35 | 3.79E-02 | 1.20 | 1.95E-01 | 1.16 | 2.75E-01 |
| **Ly9** | 1.78 | 1.28E-03 | 1.04 | 8.09E-01 | -1.18 | 3.02E-01 | 1.04 | 8.14E-01 | -1.02 | 8.94E-01 |
| **Clec4e** | 1.60 | 1.31E-03 | 1.54 | 6.35E-03 | 1.01 | 9.45E-01 | 1.03 | 8.19E-01 | 1.24 | 1.14E-01 |
| **Rin2** | 1.52 | 1.35E-03 | 1.25 | 1.01E-01 | -1.02 | 8.81E-01 | 1.08 | 5.28E-01 | 1.00 | 9.75E-01 |
| **Lcn2** | -1.03 | 9.06E-01 | 2.67 | 1.40E-03 | 1.09 | 7.23E-01 | 1.21 | 4.34E-01 | 1.25 | 3.66E-01 |
| **Rgl1** | 1.62 | 1.92E-03 | 1.07 | 6.59E-01 | -1.04 | 7.57E-01 | 1.14 | 3.74E-01 | -1.01 | 9.71E-01 |
| **Hsd17b11** | 1.54 | 1.96E-03 | 1.02 | 8.87E-01 | -1.00 | 9.73E-01 | 1.22 | 1.20E-01 | -1.08 | 5.32E-01 |
| **Zfpm1** | 1.70 | 2.72E-03 | -1.19 | 3.48E-01 | -1.18 | 3.00E-01 | -1.25 | 1.69E-01 | -1.10 | 5.63E-01 |
| **Vcam1** | 1.50 | 2.91E-03 | 1.13 | 3.92E-01 | 1.04 | 7.51E-01 | -1.03 | 7.94E-01 | 1.06 | 6.33E-01 |
| **Atp13a1** | 1.55 | 3.03E-03 | -1.09 | 5.62E-01 | 1.11 | 4.35E-01 | 1.01 | 9.25E-01 | 1.05 | 7.39E-01 |
| **Stat4** | 1.66 | 3.05E-03 | 1.20 | 3.08E-01 | -1.06 | 7.19E-01 | 1.01 | 9.65E-01 | 1.09 | 5.77E-01 |
| **Klk1b9** | -1.01 | 9.10E-01 | 1.04 | 8.07E-01 | 1.51 | 3.13E-03 | 1.11 | 4.09E-01 | 1.07 | 5.88E-01 |
| **Ms4a7** | 1.53 | 3.38E-03 | -1.11 | 4.94E-01 | 1.02 | 8.90E-01 | 1.17 | 2.33E-01 | 1.01 | 9.14E-01 |
| **Smg7** | 1.53 | 3.63E-03 | 1.21 | 2.20E-01 | 1.03 | 8.14E-01 | 1.16 | 2.65E-01 | 1.03 | 8.38E-01 |
| **Atf4** | 1.16 | 3.02E-01 | -1.10 | 5.72E-01 | 1.58 | 3.64E-03 | 1.07 | 6.52E-01 | 1.35 | 4.56E-02 |
| **Psme2** | 1.79 | 4.34E-03 | 1.34 | 1.74E-01 | 1.35 | 1.23E-01 | 1.09 | 6.44E-01 | -1.04 | 8.53E-01 |
| **Glipr2** | 1.50 | 4.36E-03 | 1.02 | 9.08E-01 | 1.25 | 9.70E-02 | 1.08 | 5.75E-01 | -1.00 | 9.76E-01 |
| **Socs3** | 1.50 | 4.45E-03 | -1.09 | 5.76E-01 | -1.16 | 2.74E-01 | -1.03 | 7.96E-01 | -1.02 | 9.03E-01 |
| **Pvrl2** | 1.62 | 4.67E-03 | 1.07 | 6.88E-01 | 1.03 | 8.72E-01 | 1.18 | 2.92E-01 | 1.13 | 4.33E-01 |
| **Tnfsf4** | 1.70 | 5.15E-03 | 1.15 | 4.92E-01 | 1.24 | 2.30E-01 | 1.07 | 7.13E-01 | 1.18 | 3.42E-01 |
| **Stfa2** | -1.46 | 1.43E-01 | 2.26 | 7.18E-03 | 1.13 | 6.25E-01 | -1.14 | 6.10E-01 | -1.22 | 4.37E-01 |
| **Nampt** | 1.54 | 8.02E-03 | -1.16 | 3.78E-01 | 1.04 | 7.80E-01 | 1.11 | 4.94E-01 | -1.10 | 5.24E-01 |
| **Zranb3** | 1.61 | 8.13E-03 | -1.26 | 2.24E-01 | -1.04 | 8.12E-01 | -1.01 | 9.37E-01 | -1.00 | 9.97E-01 |
| **Sdcbp** | 1.52 | 8.80E-03 | 1.25 | 1.80E-01 | -1.15 | 3.56E-01 | 1.27 | 1.16E-01 | 1.10 | 5.37E-01 |
| **Gpr120** | 1.58 | 8.83E-03 | -1.06 | 7.36E-01 | 1.16 | 3.67E-01 | -1.07 | 6.84E-01 | -1.03 | 8.34E-01 |
| **Sphk1** | 1.68 | 9.31E-03 | -1.14 | 5.42E-01 | -1.04 | 8.16E-01 | -1.21 | 3.20E-01 | -1.10 | 6.26E-01 |
| **Eef1b2** | -1.23 | 2.16E-01 | 1.02 | 9.09E-01 | 1.57 | 9.42E-03 | 1.05 | 7.80E-01 | -1.06 | 7.15E-01 |
| **Serpinb2** | 1.17 | 4.25E-01 | 1.75 | 1.37E-02 | -1.16 | 4.25E-01 | -1.05 | 7.88E-01 | 1.58 | 2.29E-02 |
| **Sema4d** | 1.56 | 1.49E-02 | 1.26 | 2.30E-01 | -1.10 | 5.96E-01 | -1.04 | 8.10E-01 | -1.02 | 9.30E-01 |
| **S100a9** | -1.54 | 1.59E-01 | 2.39 | 1.49E-02 | 1.23 | 5.01E-01 | 1.18 | 5.83E-01 | -1.24 | 4.82E-01 |
| **Pglyrp1** | 1.01 | 9.54E-01 | 1.86 | 1.93E-02 | 1.03 | 8.79E-01 | 1.02 | 9.39E-01 | -1.09 | 6.87E-01 |
| **Ngp** | -1.28 | 4.95E-01 | 2.61 | 2.44E-02 | 1.03 | 9.28E-01 | 1.03 | 9.31E-01 | -1.12 | 7.56E-01 |
| **Pacsin1** | 1.50 | 2.46E-02 | 1.00 | 9.91E-01 | -1.07 | 6.87E-01 | -1.02 | 8.91E-01 | -1.02 | 8.93E-01 |
| **Nes** | 1.71 | 2.84E-02 | 1.11 | 7.00E-01 | -1.02 | 9.19E-01 | 1.20 | 4.42E-01 | -1.08 | 7.31E-01 |
| **Cd177** | -1.17 | 4.27E-01 | 1.67 | 2.98E-02 | -1.07 | 7.22E-01 | -1.01 | 9.69E-01 | -1.19 | 3.93E-01 |
| **Ppap2b** | 1.57 | 3.54E-02 | 1.01 | 9.81E-01 | 1.02 | 9.40E-01 | -1.13 | 5.60E-01 | 1.07 | 7.34E-01 |
| **Mmp2** | 1.50 | 3.78E-02 | 1.22 | 3.42E-01 | -1.10 | 6.00E-01 | 1.10 | 6.15E-01 | -1.17 | 3.98E-01 |
| **Stfa1** | -1.49 | 2.72E-01 | 2.34 | 4.28E-02 | 1.11 | 7.75E-01 | -1.04 | 9.13E-01 | -1.29 | 4.81E-01 |

All genes (445) for which expression level in at least one genotype was ≥1.5-fold changed at 24 hours after WNV infection (*P* < 0.05, without correction for false discovery). Values represent the mean of three independent samples for each genotype. “Fold change” refers to the relative fold change of expression in WNV-infected mDC compared with mock-infected controls of the same genotype. DKO: *Irf3^-/-^* x *Irf7^-/-^*; TKO: *Irf3^-/-^* x *Irf 5^-/-^* x *Irf7^-/-^.*
