# Supplementary material for: IRF-3, IRF-5, and IRF-7 Coordinately Regulate the Type I IFN Response in Myeloid Dendritic Cells Downstream of MAVS Signaling
Source: PLoS Pathog. 2013 Jan 3;9(1):e1003118. doi: 10.1371/journal.ppat.1003118 (PMC3536698; doi:10.1371/journal.ppat.1003118)
Supplement: Table S2 — IFN-independent gene induction. Genes are shown for which expression level in Ifnar−/− mDC was ≥1.5-fold changed at 24 hours after WNV infection (P<0.05, without correction for false discovery). Values represent the mean of three independent samples for each genotype. “Fold change” refers to the relative fold change of expression in WNV-infected mDC compared with mock-infected controls of the same genotype. DKO: Irf3−/−×Irf7−/−; TKO: Irf3−/−×Irf 5−/−×Irf7−/−. (DOCX) [file ppat.1003118.s003.docx]

**Table S2: IFN-independent gene induction**

|  | **WT** | | **DKO** | | **TKO** | | ***Mavs^-/-^*** | | ***Ifnar^-/-^*** | |
| --- | --- | --- | --- | --- | --- | --- | --- | --- | --- | --- |
| **Gene** | **Fold change** | **P Value** | **Fold change** | **P Value** | **Fold change** | **P Value** | **Fold change** | **P Value** | **Fold change** | **P Value** |
| **Ifnb1** | 13.43 | 9.01E-18 | 4.29 | 5.04E-11 | 1.03 | 8.05E-01 | 1.05 | 6.87E-01 | 19.47 | 3.71E-19 |
| **Rsad2** | 99.45 | 2.26E-23 | 20.01 | 1.06E-17 | 1.03 | 8.41E-01 | -1.27 | 6.58E-02 | 11.90 | 6.82E-17 |
| **Cxcl10** | 73.15 | 1.13E-26 | 6.03 | 2.42E-16 | 1.05 | 5.46E-01 | 1.02 | 8.37E-01 | 3.73 | 2.48E-14 |
| **Isg15** | 58.60 | 1.27E-20 | 11.85 | 2.28E-14 | 1.00 | 9.93E-01 | -1.04 | 7.86E-01 | 6.34 | 1.27E-12 |
| **Ppp1r15a** | 2.78 | 1.41E-10 | 1.39 | 5.60E-03 | 1.40 | 2.05E-03 | 1.38 | 2.88E-03 | 3.38 | 3.62E-12 |
| **Ifit2** | 55.22 | 7.44E-23 | 15.77 | 9.33E-18 | 1.00 | 9.95E-01 | -1.01 | 9.44E-01 | 3.00 | 5.82E-10 |
| **Oasl1** | 27.11 | 6.23E-19 | 7.72 | 5.42E-13 | -1.03 | 8.18E-01 | 1.04 | 7.62E-01 | 3.42 | 2.06E-09 |
| **Il6** | 1.42 | 5.36E-07 | 1.46 | 1.10E-06 | 1.35 | 6.34E-06 | 1.05 | 3.99E-01 | 1.60 | 3.54E-09 |
| **Ifit3** | 96.42 | 5.73E-23 | 17.49 | 6.58E-17 | 1.03 | 8.09E-01 | 1.27 | 7.27E-02 | 2.99 | 6.35E-09 |
| **Trib3** | 2.00 | 1.31E-06 | 1.66 | 3.69E-04 | 2.56 | 6.58E-09 | 1.51 | 9.36E-04 | 2.40 | 2.56E-08 |
| **Ccrl2** | 4.56 | 1.51E-14 | 2.14 | 1.91E-07 | 1.14 | 1.74E-01 | -1.04 | 6.87E-01 | 1.93 | 3.12E-07 |
| **Tnf** | 1.68 | 5.11E-07 | 1.74 | 1.08E-06 | 1.05 | 5.49E-01 | 1.05 | 5.13E-01 | 1.70 | 3.63E-07 |
| **Ccl5** | 7.95 | 5.71E-12 | 3.56 | 5.83E-07 | 1.38 | 6.95E-02 | -1.05 | 7.79E-01 | 3.19 | 4.13E-07 |
| **Rgs1** | 2.70 | 9.51E-10 | 1.81 | 3.17E-05 | 1.62 | 9.86E-05 | 1.21 | 8.64E-02 | 1.96 | 1.05E-06 |
| **Chac1** | 1.48 | 6.55E-04 | 1.29 | 3.49E-02 | 1.63 | 5.87E-05 | 1.16 | 1.56E-01 | 1.87 | 1.86E-06 |
| **Ddit3** | 3.16 | 1.02E-09 | 1.83 | 1.54E-04 | 1.66 | 3.22E-04 | 1.28 | 5.62E-02 | 2.07 | 3.04E-06 |
| **Nupr1** | 1.96 | 3.34E-06 | 1.44 | 7.67E-03 | 1.43 | 4.12E-03 | 1.29 | 3.42E-02 | 1.91 | 5.95E-06 |
| **Nfkbiz** | 1.78 | 5.62E-07 | 2.06 | 7.55E-08 | 1.55 | 2.96E-05 | 1.23 | 2.66E-02 | 1.63 | 6.63E-06 |
| **Sdc4** | 1.45 | 4.51E-04 | 1.01 | 9.51E-01 | -1.05 | 6.09E-01 | -1.00 | 9.92E-01 | 1.69 | 6.98E-06 |
| **Gadd45a** | -1.01 | 9.11E-01 | 1.73 | 9.07E-04 | 1.67 | 6.18E-04 | 1.24 | 1.15E-01 | 1.66 | 6.86E-04 |
| **Gbp5** | 7.34 | 1.01E-15 | 2.79 | 1.39E-08 | 1.02 | 8.30E-01 | 1.08 | 4.77E-01 | 1.51 | 1.11E-03 |
| **Serpinb2** | 1.17 | 4.25E-01 | 1.75 | 1.37E-02 | -1.16 | 4.25E-01 | -1.05 | 7.88E-01 | 1.58 | 2.29E-02 |

Genes are shown for which which expression level in *Ifnar^-/-^* mDC was ≥1.5-fold changed at 24 hours after WNV infection (*P* < 0.05, without correction for false discovery). Values represent the mean of three independent samples for each genotype. “Fold change” refers to the relative fold change of expression in WNV-infected mDC compared with mock-infected controls of the same genotype. DKO: *Irf3^-/-^* x *Irf7^-/-^*; TKO: *Irf3^-/-^* x *Irf 5^-/-^* x *Irf7^-/-^.*
