# Supplementary material for: IRF-3, IRF-5, and IRF-7 Coordinately Regulate the Type I IFN Response in Myeloid Dendritic Cells Downstream of MAVS Signaling
Source: PLoS Pathog. 2013 Jan 3;9(1):e1003118. doi: 10.1371/journal.ppat.1003118 (PMC3536698; doi:10.1371/journal.ppat.1003118)
Supplement: Table S3 — Genes induced in IFNAR and DKO, but not TKO mDC. Genes are shown for which expression level in Ifnar−/− and DKO mDC was ≥1.5-fold changed at 24 hours after WNV infection (P<0.05), but which fell short of these cutoffs in TKO cells. Values represent the mean of three independent samples for each genotype. “Fold change” refers to the relative fold change of expression in WNV-infected mDC compared with mock-infected controls of the same genotype. DKO: Irf3−/−×Irf7−/−; TKO: Irf3−/−×Irf 5−/−×Irf7−/−. (DOCX) [file ppat.1003118.s004.docx]

**Table S3: Genes induced in *Ifnar*^-/-^ and DKO, but not TKO mDC**

|  | **WT** | | **DKO** | | **TKO** | | ***Mavs^-/-^*** | | ***Ifnar^-/-^*** | |
| --- | --- | --- | --- | --- | --- | --- | --- | --- | --- | --- |
| **Gene** | **Fold change** | **P Value** | **Fold change** | **P Value** | **Fold change** | **P Value** | **Fold change** | **P Value** | **Fold change** | **P Value** |
| **Ifit2** | 55.22 | 7.44E-23 | 15.77 | 9.33E-18 | 1.00 | 9.95E-01 | -1.01 | 9.44E-01 | 3.00 | 5.82E-10 |
| **Rsad2** | 99.45 | 2.26E-23 | 20.01 | 1.06E-17 | 1.03 | 8.41E-01 | -1.27 | 6.58E-02 | 11.90 | 6.82E-17 |
| **Ifit3** | 96.42 | 5.73E-23 | 17.49 | 6.58E-17 | 1.03 | 8.09E-01 | 1.27 | 7.27E-02 | 2.99 | 6.35E-09 |
| **Cxcl10** | 73.15 | 1.13E-26 | 6.03 | 2.42E-16 | 1.05 | 5.46E-01 | 1.02 | 8.37E-01 | 3.73 | 2.48E-14 |
| **Isg15** | 58.60 | 1.27E-20 | 11.85 | 2.28E-14 | 1.00 | 9.93E-01 | -1.04 | 7.86E-01 | 6.34 | 1.27E-12 |
| **Oasl1** | 27.11 | 6.23E-19 | 7.72 | 5.42E-13 | -1.03 | 8.18E-01 | 1.04 | 7.62E-01 | 3.42 | 2.06E-09 |
| **Ifnb1** | 13.43 | 9.01E-18 | 4.29 | 5.04E-11 | 1.03 | 8.05E-01 | 1.05 | 6.87E-01 | 19.47 | 3.71E-19 |
| **Gbp5** | 7.34 | 1.01E-15 | 2.79 | 1.39E-08 | 1.02 | 8.30E-01 | 1.08 | 4.77E-01 | 1.51 | 1.11E-03 |
| **Ccrl2** | 4.56 | 1.51E-14 | 2.14 | 1.91E-07 | 1.14 | 1.74E-01 | -1.04 | 6.87E-01 | 1.93 | 3.12E-07 |
| **Ccl5** | 7.95 | 5.71E-12 | 3.56 | 5.83E-07 | 1.38 | 6.95E-02 | -1.05 | 7.79E-01 | 3.19 | 4.13E-07 |
| **Tnf** | 1.68 | 5.11E-07 | 1.74 | 1.08E-06 | 1.05 | 5.49E-01 | 1.05 | 5.13E-01 | 1.70 | 3.63E-07 |
| **Serpinb2** | 1.17 | 4.25E-01 | 1.75 | 1.37E-02 | -1.16 | 4.25E-01 | -1.05 | 7.88E-01 | 1.58 | 2.29E-02 |

Genes are shown for which which expression level in *Ifnar^-/-^* and DKO mDC was ≥1.5-fold changed at 24 hours after WNV infection (*P* < 0.05, without correction for false discovery), but which fell short of these cutoffs in TKO cells. Values represent the mean of three independent samples for each genotype. “Fold change” refers to the relative fold change of expression in WNV-infected mDC compared with mock-infected controls of the same genotype. DKO: *Irf3^-/-^* x *Irf7^-/-^*; TKO: *Irf3^-/-^* x *Irf 5^-/-^* x *Irf7^-/-^.*
