# Supplementary material for: IRF-3, IRF-5, and IRF-7 Coordinately Regulate the Type I IFN Response in Myeloid Dendritic Cells Downstream of MAVS Signaling
Source: PLoS Pathog. 2013 Jan 3;9(1):e1003118. doi: 10.1371/journal.ppat.1003118 (PMC3536698; doi:10.1371/journal.ppat.1003118)
Supplement: Table S4 — Primers and probes used for quantitative RT-PCR. (DOCX) [file ppat.1003118.s005.docx]

**Table S4: Primers and probes used for quantitative RT-PCR:**

| **Target** |  | **Sequence** | **Ref** |
| --- | --- | --- | --- |
| **WNV-NY (E)** | Fwd | 5’-TCAGCGATCTCTCCACCAAAG-3’ | [[1](#_ENREF_1)] |
|  | Rev | 5’-GGGTCAGCACGTTTGTCATTG-3’ |  |
|  | Probe | 5’-/56-FAM/TGCCCGACCATGGGAGAAGCTC/36-TAMSp/-3’ |  |
| **WNV-MAD (E)** | Fwd | 5’-TCAGTGAGTTATCAACAAGAG-3’ |  |
|  | Rev | 5’-GGATCAGCTCTTTTCTCATTA-3’ |  |
|  | Probe | 5’-/56-FAM/TGCCCAACCATGGGAGAAGCCC/36-TAMSp/-3’ |  |
| ***Gapdh*** | Fwd | 5’-AATGGTGAAGGTCGGTGTG-3’ |  |
|  | Rev | 5’-GTGGAGTCATACTGGAACATGTAG-3’ |  |
|  | Probe | 5’-/56-FAM/TGCAAATGG/ZEN/CAGCCCTGGTG/3IABkFQ/-3’ |  |
| ***Rsad2*** | Fwd | 5’-ACACAGCCAAGACATCCTTC-3’ |  |
|  | Rev | 5’-CAAGTATTCACCCCTGTCCTG-3’ |  |
|  | Probe | 5’-/56-FAM/TGTTTGAGCAGAAGCAGTCCTCGC/3IABkFQ/-3’ |  |
| ***Ifnb*** | Fwd | 5’-CTGGAGCAGCTGAATGGAAAG-3’ | [[2](#_ENREF_2)] |
|  | Rev | 5’-CTTCTCCGTCATCTCCATAGGG-3’ |  |
|  | Probe | 5’-/56-FAM/CAACCTCACCTACAGGGCGGACTTCAAG/36-FAMSp/-3’ |  |
| ***Oas1a*** | Fwd | 5’-TGAGCGCCCCCCATCT-3’ |  |
|  | Rev | 5’-CATGACCCAGGACATCAAAGG-3’ |  |
|  | Probe | 5’-/56-FAM/AGGAGGTGGAGTTTGATGTGCTG/36-TAMSp/-3’ |  |
| ***Cxcl10*** | Fwd | 5’-AGTGCTGCCGTCATTTTCTG-3’ | [[3](#_ENREF_3)] |
|  | Rev | 5’-ATTCTCACTGGCCCGTCAT-3’ |  |
|  | Probe | 5’-/56-FAM/AGTCCCACTCAGACCCAGCAGG/36-TAMSp/-3’ |  |
| ***Ifit1*** | Fwd | 5’-GAGCCAGAAAACCCTGAGTACA-3’ | [[2](#_ENREF_2)] |
|  | Rev | 5’-AGAAATAAAGTTGTCATCTAAATC-3’ |  |
|  | Probe | 5’-/56-FAM/ACTGGCTATGCAGTCGTAGCCTATCGCC/36-TAMSp/-3’ |  |
| ***Ifit2*** | Fwd | 5’-CTGAAGCTTGACGCGGTACA -3’ |  |
|  | Rev | 5’- ACTTGGGTCTTTCTTTAAGGCTTCT-3’ |  |
|  | Probe | 5’-/56-FAM/AAAACCAAGCAATGGCGCTGGTTG/36-TAMSp/-3’ |  |
| ***Ddx58*** | Fwd | 5’-CGGCGTTGGAGATGCTAAGA -3’ |  |
|  | Rev | 5’- CAG GGC GGC ACA GAG TTT A -3’ |  |
|  | Probe | 5’-/56-FAM/CGGAGGAAGCCATGCAACATATCT/36-TAMSp/-3’ |  |
| ***Ifitm3*** | Fwd | 5’-CTCCATCCTTTGCCCTTCAG-3’ |  |
|  | Rev | 5’-TCTTCCTTGATTCTTTCGTAGTTTG-3’ |  |
|  | Probe | 5’-/56-FAM/TCCGCACCA/ZEN/TGAACCACACTTCT/3IABkFQ/-3’ |  |
| ***Ccl5*** | Fwd | 5’-CAAGTGCTCCAATCTTGCAG-3’ |  |
|  | Rev | 5’-CCTCTATCCTAGCTCATCTCCA-3’ |  |
|  | Probe | 5’-/56-FAM/TGTTTGTCA/ZEN/CTCGAAGGAACCGCC/3IABkFQ/-3’ |  |
| ***Ccl2*** | Fwd | 5’-TCAGCCAGATGCAGTTAACG-3’ |  |
|  | Rev | 5’-CTCTCTTGAGCTTGGTGACA-3’ |  |
|  | Probe | 5’-/56-FAM/ACTCACCTG/ZEN/CTGCTACTCATTCACC/3IABkFQ/-3’ |  |
| ***Nfkbiz*** | Fwd | 5'-CACTGCACTCTTCAGGTCTG-3' |  |
|  | Rev | 5'-CTCCTGCTACACATCCGAAG-3' |  |
|  | Probe | 5'-/56-FAM/TGTCTTAAA/ZEN/CTCATCCACGGGCTGG/3IABkFQ/-3' |  |
| ***Rgs1*** | Fwd | 5'-CTTCCAAAGACATTTTGACCTGT-3' |  |
|  | Rev | 5'-GAAATCGGCCAAGTCCAAAG-3' |  |
|  | Probe | 5'-/56-FAM/TCCAGAGAC/ZEN/TGAGACCACTGCATTACT/3IABkFQ/-3' |  |
| ***Trib3*** | Fwd | 5'-AAAGATGTAAAGGAGCCGAGAG-3' |  |
|  | Rev | 5'-CCACAGGCACAGAGTACAC-3' |  |
|  | Probe | 5'-/56-FAM/CTCGCTGGC/ZEN/AGGGTACACCTT/3IABkFQ/-3' |  |
| ***Ddit3*** | Fwd | 5'-GACTCAGCTGCCATGACTG-3' |  |
|  | Rev | 5'-GCGACAGAGCCAGAATAACAG-3' |  |
|  | Probe | 5'-/56-FAM/CCACCACAC/ZEN/CTGAAAGCAGAACCT/3IABkFQ/-3' |  |
| ***Chac1*** | Fwd | 5'-CCTCACATTCAGGTACTTCAGG-3' |  |
|  | Rev | 5'-GCAGCGACAAGATGCCT-3' |  |
|  | Probe | 5'-/56-FAM/ACCCCAAGT/ZEN/GCAGCCCTCAC/3IABkFQ/-3' |  |
| ***Ppp1r15a*** | Fwd | 5'-GGACAGGAGATAGAAGTTGTGG-3' |  |
|  | Rev | 5'-GATCGCTTTTGGCAACCAG-3' |  |
|  | Probe | 5'-/56-FAM/AGCCCAAGA/ZEN/CCCCAGCATGT/3IABkFQ/-3' |  |

**REFERENCES**

1. Samuel MA, Whitby K, Keller BC, Marri A, Barchet W, et al. (2006) PKR and RNase L contribute to protection against lethal West Nile Virus infection by controlling early viral spread in the periphery and replication in neurons. J Virol 80: 7009-7019.

2. Daffis S, Samuel MA, Keller BC, Gale M, Jr., Diamond MS (2007) Cell-specific IRF-3 responses protect against West Nile virus infection by interferon-dependent and -independent mechanisms. PLoS Pathog 3: e106.

3. Valbuena G, Bradford W, Walker DH (2003) Expression analysis of the T-cell-targeting chemokines CXCL9 and CXCL10 in mice and humans with endothelial infections caused by rickettsiae of the spotted fever group. Am J Pathol 163: 1357-1369.
